# Ecological features of upriver migration in Kitakami River chum salmon and their connection to aerobic thermal performance

**DOI:** 10.1093/conphys/coae087

**Published:** 2024-12-26

**Authors:** Takaaki K Abe, Takashi Kitagawa, Yuki Iino, Motohiro Ito, Katsufumi Sato

**Affiliations:** Department of Marine Bioscience, Atmosphere and Ocean Research Institute, The University of Tokyo, 5-1-5, Kashiwanoha, Kashiwa, Chiba 277-8564, Japan; Department of Living Marine Resources, Atmosphere and Ocean Research Institute, The University of Tokyo, 5-1-5, Kashiwanoha, Kashiwa, Chiba 277-8564, Japan; Department of Marine Science, College of Bioresource Science, Nihon University, 1866 Kameino, Fujisawa, Kanagawa 252-0813, Japan; Department of Living Marine Resources, Atmosphere and Ocean Research Institute, The University of Tokyo, 5-1-5, Kashiwanoha, Kashiwa, Chiba 277-8564, Japan; Department of Natural Environmental Studies, Graduate School of Frontier Sciences, The University of Tokyo, 5-1-5, Kashiwanoha, Kashiwa, Chiba 277-8564, Japan; Department of Living Marine Resources, Atmosphere and Ocean Research Institute, The University of Tokyo, 5-1-5, Kashiwanoha, Kashiwa, Chiba 277-8564, Japan; Department of Applied Biosciences, Faculty of Life Sciences, Toyo University, 1-1-1 Izumino, Itakura-machi, Oragun, Gunma 374-0193, Japan; Department of Marine Bioscience, Atmosphere and Ocean Research Institute, The University of Tokyo, 5-1-5, Kashiwanoha, Kashiwa, Chiba 277-8564, Japan

**Keywords:** Absolute aerobic scope, data logger, oxygen- and capacity-limited thermal tolerance, Pacific salmonid, radio telemetry

## Abstract

The physiological performance of ectotherms is influenced by temperature, raising concerns about the impact of global warming on ectotherms. Understanding the relationship between ecologically relevant temperatures and the physiological performance of ectotherms provides a basis for assessing their resilience to changing environments. Absolute aerobic scope (AAS) is a functional metric of the thermal performance of aquatic ectotherms. The thermal profile of chum salmon (*Oncorhynchus keta*) returning to the Kitakami River, Japan, in early October has already been explored in a previous study; however, the ecological aspects of their upriver migration (e.g. spawning site, migratory duration and experienced temperature) and their connection to AAS thermal profiles are not fully understood. To address this gap, we released 53 marked chum salmon throughout the spawning season (October–November), of which 48 were tracked using radio telemetry. Over 3 years, 18 were successfully tracked to their spawning sites, and 13 were tracked partway. The longest track was 93 km. The spawning sites of Kitakami River chum salmon depended on migration timing, with earlier run salmon tending towards upriver sites. Chum salmon returning in October spawned in the middle basin, typically requiring >5 days to reach the spawning sites, whereas those returning in November spawned in the lower sections in 1–3 days. Comparing the estimated thermal occupancy of migrating salmon with the published AAS profile, we found that Kitakami River chum salmon in early October spent almost all of their time within the optimal temperature window for AAS and tended to be below the peak temperature of AAS. Our findings provide a basis for the ecological features of migrating chum salmon in rivers and shed light on their aerobic thermal performance in the natural environment.

## Introduction

The physiological performance of ectotherms is largely influenced by temperature through thermodynamic effects on metabolism. The metabolic rate of ectotherms, generally assessed by measuring oxygen consumption rates, rises or sometimes falls sharply with temperature; therefore, there are growing concerns about the impact of advancing global warming on ectotherms (Schulte, 2015). To predict the effects of future warming, it is crucial to understand the thermal adaptation of ectotherms in their current environment and the influence of temperature changes. The mechanistic basis of thermal adaptation is typically described by the thermal performance curve (Fry, 1947; Fry and Hart, 1948), where the performance increases with temperature up to an optimal point (referred to as the optimum temperature, ${T}_{\mathrm{opt}}$) and subsequently declines. Ectotherms generally have a thermal performance curve that is adapted to their thermal niche, with an optimal temperature range (known as ${T}_{\mathrm{opt}}$ window) (Huey and Stevenson, 1979; Huey and Kingsolver, 1989). The concept of oxygen- and capacity-limited thermal tolerance (OCLTT) is widely recognized as a framework for understanding thermal adaptation in aquatic ectotherms (Pörtner and Farrell, 2008). OCLTT posits that the absolute aerobic scope (AAS), which is the difference between minimum and maximum (aerobic) metabolic rates, is a functional metric for thermal performance because most biological processes (e.g. swimming, digestion, maturation) are accomplished through aerobic metabolism.

The OCLTT concept has been examined in spawning adults of Pacific salmonids during their freshwater migration to spawning grounds (Clark *et al.,* 2011; Eliason *et al.,* 2011; Eliason *et al.,* 2013; Raby *et al.,* 2016; Abe *et al.,* 2019). Pacific salmonids are anadromous and generally exhibit fidelity to their spawning grounds, resulting in a variety of life history traits at the species- and population-levels, even within the same river system (Groot and Margolis, 1991). The migration timing of spawning adults varies across species and/or populations, exposing them to distinct thermal conditions (Eliason *et al.,* 2011; Eliason *et al.,* 2013; Abe *et al.,* 2019). These observations suggest local adaptation of thermal performance, and studies have measured the AAS of spawning adults of Pacific salmonid species at various water temperatures. Previous studies have revealed species- or population-specific performance curves and an approximate relationship to empirical temperatures (Clark *et al.,* 2011; Eliason *et al.,* 2011; Eliason *et al.,* 2013; Raby *et al.,* 2016; Abe *et al.,* 2019), reaffirming the important role of aerobic performance in the thermal adaptation of fish.

Chum salmon (*Oncorhynchus keta*) have the most extensive natural geographic distribution amongst all Pacific salmon species, with spawning regions ranging across the North Pacific Ocean from Asia to North America and extending into the Arctic Ocean (Salo, 1991). They predominantly spawn in the middle reaches of rivers and quickly reach their spawning grounds compared with other Pacific salmon (Salo, 1991). Despite previous studies on the upriver movements of Pacific salmonids (Rand *et al.,* 2006; Martin *et al.,* 2015) and thermal profiles of AAS (Lee *et al.,* 2003; Clark *et al.,* 2011; Eliason *et al.,* 2011; Eliason *et al.,* 2013; Raby *et al.,* 2016), the upriver migration and its connection to the aerobic performance of chum salmon remain poorly understood. Knowledge of the upriver migration of chum salmon is fragmented and limited to short distances (Akita *et al.,* 2006; Makiguchi *et al.,* 2007; Nobata *et al.,* 2022) and the middle sections in rivers (Aruga *et al.,* 2009). Similarly, information regarding thermal performance is obtained from a single study of two populations in Japan (Abe *et al.,* 2019). Even in Japan, where chum salmon have high commercial value, chum salmon behaviour and spawning habitats in rivers have not been explored because the majority of spawning adults returning to Japanese waters have been considered to be of hatchery origin because of stock enhancement programs. However, a recent study has reported numerous naturally spawning chum salmon in non-enhanced rivers, indicating the possible importance of wild populations for the entire Japanese stock (Miyakoshi *et al.,* 2012). As a result, interest in the behaviour and habitat of wild chum salmon in Japan has increased.

**Figure 1:**
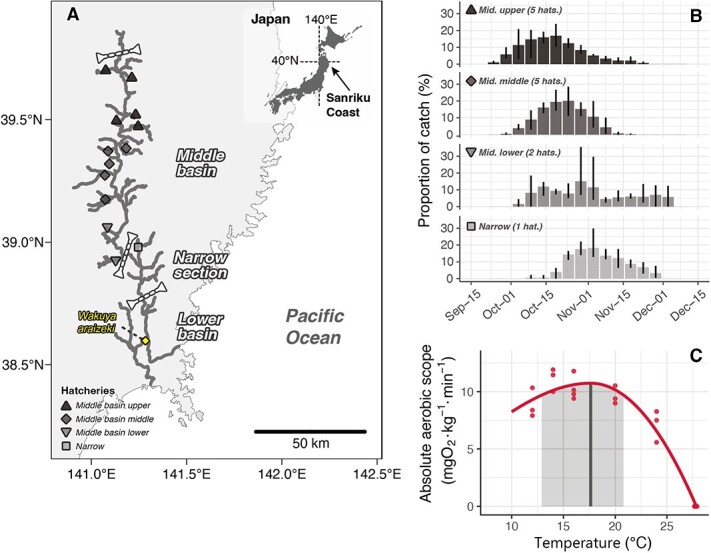
Study map (A), chum salmon catch information in the Kitakami River system (B) and thermal performance curve of chum salmon in the Kitakami River (C). (A) The Kitakami River is located in the Sanriku coastal area of Japan. The Kitakami River is partitioned into several sections, and salmon runs are mainly observed in the lower basin, narrow section and middle basin. The white dashed lines represent the threshold between the lower basin, narrow section and middle basin. All salmon used in this study were captured at Wakuya Araizeki (labelled diamond), an overfall weir. Thirteen hatcheries in the Kitakami River system are denoted by triangles, diamonds, and squares. The symbol shapes and colour intensities indicate hatchery classification, with the upper side (Mid. upper, 121–157 km from the overfall weir), middle (Mid. middle, 84–113 km from the overfall weir) and lower side (Mid. lower, 57–67 km from the overfall weir) in the middle basin and in the narrow section (Narrow, 48 km from the overfall weir). (B) The bars show the average proportion of adults returning out of the total number of adults caught at each hatchery from 2013 to 2016 in 5-day periods. The error bars denote the minimum and maximum proportion values over 4 years. (C) Thermal performance curves of the AAS in Kitakami River chum salmon reported by Abe *et al.* (2019). The peak of the curve and sub-optimal temperature range were defined as the optimal temperature for the absolute aerobic scope (${T}_{\mathrm{optAAS}}$, vertical bold line) and the optimal temperature window (shaded area, defined by 90% of AAS maxima).

The Sanriku coastal area in Japan serves as one of the southernmost spawning regions for chum salmon, with adults returning from autumn to winter (Tanaka *et al.,* 2000) (Fig. 1a). The Kitakami River, located along the Sanriku coast, is one of the longest rivers in Japan, where spawning adults return between October and November (Fig. 1b). The spawning redds are generally found in the main channel and tributaries downstream from the middle basin. The AAS thermal performance curve for Kitakami River chum salmon has been described using salmon captured in the lower basin in the first half of October (Fig. 1c) (Abe *et al.,* 2019), where the peak temperature (optimal temperature of AAS, ${T}_{\mathrm{optAAS}}$) was 17.6°C. The optimal temperature window for AAS (${T}_{\mathrm{optAAS}}$ window), determined as the maximum and minimum temperatures over which AAS maintains >90% of the maximum AAS (${\mathrm{AAS}}_{\mathrm{max}}$), was 12.8–20.8°C, and the window was compared to the river temperatures during October. However, the actual thermal occupancy of Kitakami River chum salmon captured in the first half of October during their upriver migration is not fully understood because of limited knowledge regarding the salmon’s upriver migration ecology, including the location of spawning sites in the river and the time required for migration. The salmon catch information from the hatcheries located in the middle basin to the narrow section indicates that catches peak earlier for upriver hatcheries and later for downriver hatcheries, suggesting that the location and time taken to reach the site vary depending on the timing of migration. To gain a better understanding of the thermal ecology of Kitakami River chum salmon, particularly those returning in early October, it is crucial to investigate the characteristics of their upriver migration, migration speed and duration from which they reach their spawning sites throughout the spawning season.

The aim of this study was to elucidate the spawning location, upriver migration speed and duration of Kitakami River chum salmon throughout the spawning season and to examine their relationship with the thermal performance of AAS. To investigate the upriver migration of Kitakami River chum salmon, 53 salmon were marked and released for recapture between October and November over 3 years. Of the 53 salmon, 48 were equipped with electronic tags, and their upriver movements were tracked using radio telemetry. Based on the tracking data, we estimated the migration duration, migration speed and thermal conditions of the population during upriver migration. Finally, we compared the thermal performance of AAS with the migration temperatures experienced by salmon entering the Kitakami River in the first half of October to gain insights into the aerobic thermal performance of these salmon in their natural environment.

## Materials and Methods

### Field experiments and data collection

Fieldwork was conducted in November 2016, October–November 2017 and October 2021 in the Kitakami River system. The Kitakami River is 249 km long with a basin area of 10 150 ${\mathrm{km}}^2$. This river is divided into five sections based on the environmental characteristics of the river, and spawning redds of chum salmon are found in the main channel and tributaries of three sections: the lower basin, narrow section and middle basin (Table 1). In the lower basin and narrow section, rice fields and unused land occupied the area around the river, whereas urban areas occupied the middle basin. The width and slope of the river in the three sections ranged from 80 to 350 m and 0.08 to 1.00 $\mathrm{m}\cdotp{\mathrm{km}}^{-1}$, respectively (Table 1). Chum salmon captured upstream from the narrow section of this river are used for hatchery-enhanced programs whilst most of those captured in the lower basin are usually used for food. In the present study, we used chum salmon captured at Wakuya Araizeki, an overfall weir ~35 km from the mouth of the Kyu-Kitakami River, by local fishermen using dip nets (Fig. 1). There were no structures between the weir and the middle basin of the river that could have obstructed salmon migration.

**Table 1:** Characteristics of each channel in the Kitakami River. Distance indicates the distance from the release point of the salmon tagged in this study, and the ranges in parentheses indicate the distance from the river mouth

**Section**	**Distance (km)**	**Elevation (m)**	**Slope (m** $\cdotp$**km** ^**−1**^**)**	**Width (m)**
Middle basin	54–157 (89–192)	11–130	0.27–1.00	80–200
Narrow section	21–54 (81–114)	4–11	0.13–0.27	80–200
Lower basin	0–21 (35–56)	1–4	0.08–0.13	200–350

### Tagging

A total of 53 chum salmon [mean (±SD) fork length 69.7 (±5.6) cm; mean (±SD) body mass 3.6 (±1.0) kg] were tagged for mark–recapture and active tracking (November 2016: $n=8$, October and November 2017: $n=25$ and $n=6$, respectively, October 2021: $n=14$) (Table 2). Plate tags with contact information, very high frequency (VHF) radio transmitters (MM130B: Advanced Telemetry Systems, Isanti, MN, USA; 16 mm diameter, 60 mm length and 16 g in air) and three types of animal-borne data loggers for water temperature measurements (W-PD3GT: Little Leonardo, Tokyo Japan; 21 mm diameter, 116 mm length and 60 g in air; ORI-PD3GTC: Little Leonardo, Tokyo, Japan; 16 mm diameter, 74 mm length and 29 g in air; Axy-Depth, TechnoSmArt, Rome, Italy; 12 mm width, 31 mm length, 11 mm height and 6.5 g in air) were used. Plate tags were attached to all salmon before release on the lateral side below their first dorsal fin. VHF radio transmitters were attached to 25 fish, data logger packages including the VHF transmitter were attached to 23 fish and 5 fish were attached with only plate tags. The five fish with only plate tags were released in October 2017. The data logger packages were created on the basis of a previous study (Nakamura *et al.,* 2015). The packages consisted of a single-unit foam buoy, a time-release mechanism, a VHF transmitter and a data logger. The instrument packages were automatically detached from each salmon after 2–10 days of deployment by cutting the plastic tie connected to the salmon via a programmed release mechanism. After detachment, the tag packages floated to the surface, and those drifting in the river were located and retrieved using VHF transmitters. All data loggers recorded the temperature at 1 Hz. The temperature resolution of W-PD3GT was 0.1°C, that of ORI-PD3GTC was 0.1°C and that of Axy-Depth was 0.01°C. The temperature accuracy of the data loggers was confirmed to be within 0.2°C by immersing them in a water-filled bucket alongside a high-accuracy temperature logger (DEFI2-T: JFE Advantech, Kobe, Japan; 0.001°C resolution; $\pm$0.01°C accuracy).

**Table 2:** Mean ± SD fork length and body mass of chum salmon released over 3 years. The ranges in parentheses represent the minimum to maximum values

	***n***	**Fork length (cm)**	**Body mass (kg)**
**2016**			
November	8	67.7 ± 5.8 (58.4–75.2)	3.3 ± 1.0 (2.1–4.8)
**2017**			
October	25	69.2 ± 4.9 (59.2–81.1)	3.5 ± 1.0 (2.4–6.6)
November	6	76.6 ± 3.1 (71.8–80.6)	4.6 ± 0.7 (3.6–5.7)
**2021**			
October	14	68.9 ± 6.0 (59.0–77.0)	3.6 ± 0.9 (2.1–4.9)
**Total**	**53**	69.7 ± 5.6 (58.4–81.1)	3.6 ± 1.0 (2.1–6.6)

Tagging was conducted based on a previous tracking study of spawning adult chum salmon (Makiguchi *et al.,* 2008). Prior to tagging, all fish were anaesthetised using FA100 (eugenol, 107 $\mathrm{mg}\cdotp{\mathrm{ml}}^{-1}$; Tanabe Seiyaku Co. Ltd, Osaka, Japan) at a concentration of 0.5 $\mathrm{ml}\cdotp{\mathrm{l}}^{-1}$. Under anaesthesia, fish were measured for fork length (FL) and body mass (BM), and VHF tags or logger packages were secured to the lateral side in front of their first dorsal fins using cable ties. After the operation, fish recovered from anaesthesia in a 250-l water tank filled with river water before being transported to a location upstream of the overfall weir for release (Fig. 1a).

### Active tracking

Active tracking of 48 released fish equipped with VHF tags ($n=25$) or tag packages ($n=23$) was performed daily throughout the experimental period (2016: 7–29 November; 2017: 1 October–8 November; 2021: 1–29 October). The VHF signal from each fish was detected using a receiver (FT-818ND, Yaesu Musen Co. Ltd, Tokyo, Japan), a monopole antenna mounted on the roof of a vehicle and a mobile four-element Yagi antenna. The monopole antenna was omnidirectional, and its maximum detection range was ~1 km radius. The four-element Yagi antenna was directional, and the maximum detection distance depended on the condition, but the VHF signal from underwater could be detected at least 5 km without any obstruction. The maximum distance between the Kitakami River and the riverside road was ~400 m in the lower basin and the narrow section of the Kitakami River, a distance sufficient for radio waves to be detected from shore. In some sections of the middle basin, it was difficult to access the riverside by vehicle because the urban area occupied the middle basin. For these cases, the signals were searched from bridges across the river or mountains near the river. During the experimental period, a typhoon passed through the Kitakami River in the afternoon of 22 October 2017, causing high water levels and flow. Until the water levels and currents subsided (2 days after the typhoon had passed), the VHF detection was conducted from bridges over the river or mountains near the river.

The active tracking started from the release point because the survey base was located near the release point, moving upriver along the riverside road. The search was concentrated on a 40 km area from the location where salmon had been observed the preceding day, as salmon are known to migrate upriver, typically within a migration range of a few to 40 km per day (Makiguchi *et al.,* 2008; Aruga *et al.,* 2009). When a VHF signal was detected, the radio signal sensitivity (gain) of the receiver was reduced to narrow the positional range. After refining the positional range, we recorded coordinates using a smartphone (2016–17: iPhone 8, A1863, Apple Inc., Cupertino, USA; 2021: iPhone SE 2nd Gen, A2275, Apple Inc., Cupertino, USA). The GPS positioning accuracy of the smartphone was enhanced by a Bluetooth GPS receiver (Garmin GLO Add-on GPS Receiver, Garmin Ltd, Olathe, USA), providing an accuracy radius of ~3 m. The accuracy of tracking points with the monopole antenna was ~100 to 200 m radius based on the results of the calibration by placing the VHF transmitter underwater. In instances where vehicle access to the riverside was difficult, the location was narrowed to within 200 m by walking along the riverbank and using the Yagi antenna for three-point positioning.

On the day of release, we checked the VHF signal transmitted from each salmon at the release point every 2–3 h until sunset or the start of upriver movement, as a previous study reported that the effect of anaesthesia could last up to 6 h (Hayashida *et al.,* 2013). Some individuals exhibited staying behaviour near the release point shortly after being released. From the following day, surveys began each morning (~7 a.m.) and continued until sunset (~6 p.m.) if any of the tracked salmon remained unlocated. If a salmon entered a tributary or stopped movement, its detailed location (within a radius of ~1–20 m) was identified with a four-element Yagi antenna to confirm whether it was alive and whether it had begun reproductive behaviour. If the salmon was alive, the position would typically move within several to 10 m during the search or by the next day, so if the signal position did not move when approaching or the next day, it was judged to be dead. The position of salmon after stopping migration due to the onset of reproductive behaviour was confirmed every 2–5 days for a period of 5–12 days.

### Classification of the fate of tracked salmon

**Figure 2:**
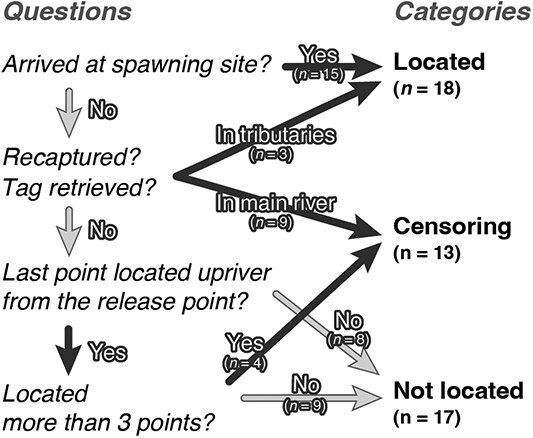
Flowchart illustrating classification of tracking results. According to the criteria (the left column), each salmon’s tracking data was categorized into three groups (right column): located (located at spawning site), censoring (censoring of tracking) and not located. The numbers listed beneath each category denote the number of salmon assigned to that classification.

The tracking results were classified into three categories, ‘located at spawning site’, ‘censoring of tracking’ and ‘not located’ (Fig. 2). ‘Located at spawning sites’ was determined in two ways: when tracked salmon arrived at the spawning site or when tracked salmon were recaptured by local fishermen in tributaries (Fig. 2). Arrival at the spawning site by tracked salmon was considered when they ceased upriver migration and remained at the site for >2 days, or entered tributaries. Whilst Atlantic salmon (*Salmo salar*) have been reported to migrate several to a dozen kilometres not only upstream but also downstream around the spawning site (defined as ‘search’ phase in Øklamd *et al.* (2001)), we observed chum salmon after they ceased migration and found that they did not migrate >1 km once they stayed there for 2 days, and they never left a tributary once they entered. When the tracked chum salmon stopped migrating, we approached the salmon using the portable Yagi antenna close enough to visually observed the reproductive behaviour (digging and courtship behaviour). It was not possible to directly observe the reproductive behaviour in all individuals that stopped upriver migration due to the river’s wide width, the high velocity of the stream centre or the presence of too many other salmon, making it difficult to identify tracked individuals. However, under such circumstances, other salmon exhibiting reproductive behaviour and spawning redds were observed. When the VHF signal from the tracked individual was detected from a spawning group at the location, the individual was classified as ‘located at spawning ground’.

‘Censoring’ represents a condition in which the value of a measurement or observation is only partially known. Tracked salmon whose upriver migration could be traced up to the halfway point but could not identify the spawning site (or tributary) were considered to have been censored from tracking. Therefore, ‘censoring of tracking’ was determined for individuals that began migrating upriver and were tracked at more than three points, including the release point (for a minimum of 2 days after release), or were recaptured in the main river (Fig. 2). For salmon equipped with data logger packages, if the tags were automatically detached via a time-release system or dislodged by a river structure (e.g. wood and aquatic plants) during tracking, these individuals were classified under this category.

Individuals whose upriver movements could not be recorded or were considered to have en route mortality shortly after release were classified as ‘not located’. This category included individuals equipped with data logger packages that remained downriver of the release point until the tag packages were released, and their tag packages were recovered downstream of the release point.

## Upriver Migration Analysis

All data analyses, including statistical procedures, were performed in R (R Core Team (2024), v4.4.1). All statistical models were compared using Akaike’s information criteria (AIC) and Bayesian information criteria (BIC) to determine a parsimonious model.

### Migration distances of chum salmon

The migration distances of chum salmon up the river were determined by projecting GPS coordinates obtained from active tracking onto the river line using the *riverdist* package in R (Tyers (2024), v0.16.3). The river shape file used for the calculation was downloaded from digital national land information supplied by the Ministry of Land, Infrastructure, Transport and Tourism of Japan (MLIT) and was converted into a universal transverse mercator (UTM) coordinate system. Similarly, the GPS points obtained from active tracking were converted to the UTM coordinate system and projected onto the nearest point on the river line using the ‘xy2segvert’ function. The migration distance of each tracking point was then computed from the projected points using the ‘riverdistancemat’ function.

### Durations and distances to reach spawning site

To examine whether the duration and distance to the spawning site varies depending on the start timing of salmon migration, survival analysis was conducted using *survival* package in R (Therneau (2024), v3.6.4). In this analysis, ‘arrival at spawning site’ was treated as the event of interest, and ‘censoring of tracking’ was considered as censored observation and both ‘time-to-event’ and ‘distance-to-event’ were modelled. Kaplan–Meier (KM) curve was used to estimate the time and distance for salmon to reach the spawning site. A preliminary Cox regression analysis (proportional hazards analysis) was performed to assess the effect of migration timing on the rate of ‘migrating’ (or ‘not arriving at spawning site’) and confirmed that earlier returning salmon migrate upriver and take longer to reach the spawning site (Supplementary Table S1). However, it was anticipated that the sample size would not be large enough for KM analysis with detailed classification, such as division into the first and second halves of October and November (four groups total) (Supplementary Table S1). Therefore, KM analysis was performed separately for the October- and November-returning salmon. Here, time was determined as the time it took to arrive at the spawning site (or tributaries), and distance was determined as the position on the river line projected from the GPS tracking point (details provided in ‘Migration distances of chum salmon on each day’ section). Some salmon classified as ‘censoring of tracking’ were found as carcasses several weeks after release. If the carcass position was upriver from the last tracking point, the carcass position was used as the censored data for the KM analysis of distance. Differences in the migrating rates between months were examined using the log-rank test.

To estimate the relationship between migratory duration and distance, a linear model (‘lm’ function of *base* package) was used. The tracking data categorized as ‘located at spawning site’ and ‘censoring of tracking’ were used for the analysis. Segment regression analysis was performed for the linear model using ‘segmented’ function from the *segmented* package in R (Muggeo (2008), v2.1.2) because of the observed tendency for migratory speed to decrease as salmon moved upriver. In addition to comparing AIC and BIC between models, if the model with a break point showed the lowest AIC and BIC, a Davies test was conducted on the segmented model to validate the break point (Muggeo, 2008).

### Daily migratory speed of each salmon

It was observed that once chum salmon began their upriver migration, they tended to continue moving upriver until arriving at their spawning site in the daily time–scale data. Therefore, fine-scale migratory speed can provide a basis for estimating the (minimum) migration duration of chum salmon arriving at spawning sites in the Kitakami River. To estimate the fine-scale migratory speed of each chum salmon, a generalized additive mixed model (GAMM) was applied to the relationship between the distance from the release site and the time after the start of upriver migration because this relationship was assumed to be non-linear. In this analysis, data with long sampling intervals were removed to assess the relationship between distance and time on a one- to a few-day timescale. Specifically, data points with intervals >3 days were excluded because VHF signals could not be detected from each salmon daily, resulting in tracking intervals >3 days for some individuals. Some individuals showed staying behaviour soon after release, lasting up to 8 days, and data on the initial stay period were also omitted from the analysis. The omitted data length in 14 of 18 salmon was <24 h, but for four, it was ~3 days. Two of the four salmon were clearly prevented from migrating due to increased discharge from the typhoon that passed through on 22 October 2017, whilst the reason for the remaining two was unclear.

Eighteen salmon were selected from those categorized into ‘located spawning site’ and ‘censoring of tracking’ based on the following criteria. Individuals whose spawning sites were within the lower basin (<30 km from release point) were excluded from this analysis ($n=4$) because they arrived there and began their reproductive behaviour within a day after the start of their upriver migration, making their migratory speeds less accurately measurable. Similarly, logger package-equipped salmon whose tags surfaced in the lower basin were also excluded ($n=3$). Additionally, individuals with only two tracking points because of the data thinning were also excluded from the analysis ($n=6$).

Smoothing splines (thin plate regression spline) were used for basic functions, and individual effects were tested as random effects for both the basic functions (i.e. random slope) and intercepts because the migratory speed and the start points of upriver migration varied between individuals. The effects of year (2016, 2017, 2021), month (October, November) and half of month (first/second half of October/November) were also tested preliminary. However, incorporating these factors as random effects into GAMM increased AIC and BIC (Supplementary Table S2). Additionally, no clear effects of year and month were observed in the time–distance relationship (Supplementary Fig. S1), so these factors were excluded from the analysis. Following the determination of the random effects model with lowest AIC and BIC, the autocorrelation structure of the residual was assessed because the tracking data were time–series data. For autocorrelation effects, autoregressive (AR) process was examined, and the model with the autocorrelation structure that resulted in the lowest AIC and BIC values was selected. Restricted maximum likelihood (REML) was used to calculate AIC and BIC for model comparisons with different random effects and autocorrelation structures (Zuur *et al.,* 2009). For the model comparison to consider fixed effects, the GAMMs were estimated using maximum likelihood (ML). All GAMM estimations were performed using ‘gamm’ function of *mgcv* package in R (Wood (2011), v.1.9.1).

## Analysis of Aerobic Performance of Upriver-Migrating Salmon

### Estimation of experienced temperature and AAS performance in tracked salmon

To evaluate the thermal performance of the tracked salmon, the temperatures they experienced were examined. Actual measurements of experienced temperature were obtained from salmon equipped with data loggers, whilst the experienced temperature of those with VHF transmitters was estimated from the river temperature record of the Kitakami River. The Kitakami River temperature has been automatically monitored by MLIT since 2002, with a resolution of 0.1°C on an hourly basis. For the analysis, river temperature records collected at a monitoring site 6 km upstream of the release point were used. A comparison between the water temperature records in the Kitakami River and those retrieved from 13 of 23 individuals equipped with data logger showed that the Kitakami River temperature records did not differ much from the temperature experienced by fish (<1°C; $\mathrm{mean}\pm \mathrm{SD}=-0.1\pm 0.7$°C, $n=1130$, Supplementary Fig. S3). This observation was consistent with a previous study (Donaldson *et al.,* 2009). In this comparison, the data recorded by the data logger were downsampled to hourly intervals to align sampling rates for river temperature. Further, data were excluded from individuals whose tags could not be retrieved ($n=1$), with a recording period of less than half a day valid for analysis ($n=1$), those that did not migrate upriver from the release site and presumably died there because of a typhoon ($n=2$), those whose tags were retrieved but failed to record data due to logger malfunction ($n=3$) and those whose tags were caught on river structures (e.g. wood or aquatic plants) near the release point and subsequently dislodged within a few days ($n=3$).

Comparisons of ${T}_{\mathrm{optAAS}}$ window and thermal performance estimates were performed for individuals migrating upriver of the narrow section ($n=21$). For the analysis, water temperatures recorded by data loggers were used for salmon with data logger packages ($n=8$), and the Kitakami River temperature records were used for salmon equipped only with VHF tags ($n=10$) and for those with data logger packages that experienced data logger malfunction ($n=3$). The water temperature recorded by the data logger was downsampled to hourly intervals to align sampling rates for the river temperature in this analysis. In order to evaluate the aerobic performance of chum salmon migrating upriver, the AAS was estimated using the thermal performance curve determined in a previous study (Abe *et al.,* 2019). The thermal performance curve of AAS had been estimated with a two-part performance curve after Deutsch *et al.* (2008) and Payne *et al.* (2016):


$$ AAS(T)=\left\{\begin{array}{@{}ll}S{e}^{-{\left(\frac{T-{T}_{\mathrm{optAAS}}}{2\sigma}\right)}^2}& \left(T\le{T}_{\mathrm{optAAS}}\right)\\{}S\left(1-{\left(\frac{T-{T}_{\mathrm{optAAS}}}{T_{\mathrm{optAAS}}-{T}_{\mathrm{crit},\max }}\right)}^2\right)& \left(T>{T}_{\mathrm{optAAS}}\right)\end{array}\right., $$

where $T$ is temperature, ${T}_{\mathrm{optAAS}}$ is the temperature at which AAS is maximized, $\sigma$ is the standard deviation (SD) of the normally distributed half of the curve, ${T}_{\mathrm{crit},\max }$ is the highest temperature at which AAS is zero and $S$ is a scalar equal to the maximum of AAS. The parameters were cited from Abe *et al.* (2019) (Supplementary Table S3). The AAS of each chum salmon was estimated by substituting empirical water temperatures into the AAS thermal performance curve.

### Estimation of AAS thermal performance of Kitakami River chum salmon during upriver migration

The thermal occupancy of chum salmon returning to the Kitakami River was estimated using the river temperature records. The examination period of thermal occupancy spanned 6 years, from 2016 (the start year of this study) to 2021 (the final year). The river temperature records were downloaded from the MLIT database mentioned above. The mean, SD and min–max range of the 6-year river temperature record were tabulated and compared with the ${T}_{\mathrm{optAAS}}$ window. We also examined the temperatures experienced by salmon that began migration in the first half of October, as the thermal performance of AAS in the previous study was estimated from the salmon captured in early October (Abe *et al.,* 2019). The spawning site was assumed to be 100 km upstream of the release point, which generally corresponded to the midpoint of the middle basin in the Kitakami River, and the time to arrival at the spawning site was calculated from the relationship between the number of days after the start of migration and the distance modelled by GAMM. The departure dates were divided into four sections (1, 6, 11 and 16 October) to estimate the water temperatures that could be experienced in 2016–21. The estimated experienced temperatures were converted into AAS and compared to the maximum value. The proportions of time spent within the ${T}_{\mathrm{optAAS}}$ window were estimated, and the proportions belonging to the high or low side of the ${T}_{\mathrm{optAAS}}$ window were examined.

### Ethics statement

All experimental procedures were performed in accordance with the guidelines of the Animal Ethics Committee of the University of Tokyo, and the study protocols were approved by the same committee (P16–7 and E21–14).

## Results

### Field-tagging study

#### Mark–recapture and tag retrieval

**Figure 3:**
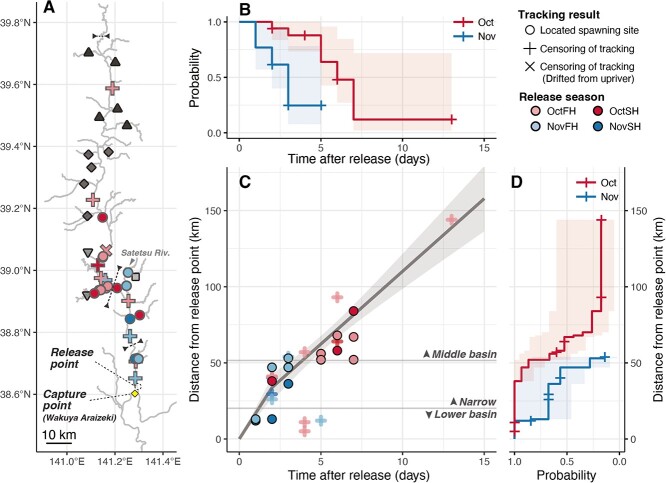
Tracking results of chum salmon migration in the Kitakami River. (A) Last tracking points of chum salmon. Filled circles and crosses represent the spawning (i.e. ‘located at spawning site’) and last tracking points (i.e. ‘censoring of tracking’), respectively. The X symbol indicates individuals that were found flowing from upstream. Filled colours denote the released season. The hatcheries in the Kitakami River are denoted by dark-coloured symbols (upper triangles: upper side of middle basin, diamonds: middle part of middle basin, lower triangles: lower part of middle basin, square: narrow section). The dashed lines represent the threshold between the lower basin, narrow section and middle basin. (B–D) Tracking durations (B) and distances (D) of the last point from the release point, and the relationship between them (C). The shapes of the plots represent the tracking results, and their meanings are the same as in (A). Tracking durations and distances are represented in KM curves by months (i.e. October and November). The transparent areas in (B) and (D) denote the 95% CI of the KM curves. The bold line and transparent area in (C) represent the segment regression line and the 95% CI, respectively.

A total of 53 salmon were released over 3 years, of which 48 were attached with electronic tags (VHF: $n=25$; logger package: $n=23$) and five were attached with plate tags only. The five salmon attached only to plate tags were not recaptured, but three fish with electronic tags were recaptured by local fishermen (Table 3). Two salmon were recaptured in a tributary (the Satetsu River) 40 km upriver from the release point and released within a few days. One salmon was recaptured in the main channel 144 km after 13 days of release (Fig. 3a and c).

**Table 3:** Summary of active tracking results for chum salmon equipped with VHF tags or data logger packages across different months and years. Tracked chum salmon (using VHF tags and logger packages) were categorized into three categories: located spawning sites, censoring of tracking and not located. The values represent the number of fish in each category, with the numbers in parentheses indicating the breakdown of individuals equipped with VHF tags (left part) versus those equipped with data loggers (right part). ‘NA’ indicates Not Applicable and does not apply to salmon equipped with VHF tags

	**Oct. ‘16**	**Oct. ‘17**	**Nov. ‘17**	**Oct. ‘21**	**Total**
**Total (VHF, Logger package)**	**8 (0, 8)**	**20 (14, 6)**	**6 (3, 3)**	**14 (8, 6)**	**48 (25, 23)**
**Located spawning site**	**4 (0, 4)**	**7 (6, 1)**	**4 (2, 2)**	**3 (2, 1)**	**18 (10, 8)**
Arrival at spawning site	4 (0, 4)	7 (6, 1)	2 (2, 0)	2 (2, 0)	15 (10, 5)
Recaptured at tributary			2 (0, 2)		2 (0, 2)
Tag retrieval at tributary				1 (0, 1)	1 (0, 1)
**Censoring of tracking**	**3 (0, 3)**	**4 (1, 3)**	**2 (1, 1)**	**4 (2, 2)**	**13 (4, 9)**
Loss of VHF signal after several days of tracking		1 (1, 0)	1 (1, 0)	1 (1, 0)	3 (3, 0)
Recaptured in the main river				1 (1, 0)	1 (1, 0)
Premature release of tag packages (tag retrieval in the main river)				1 (NA, 1)	1 (NA, 1)
Scheduled time-release of tag packages (tag retrieval in the main river)	3 (NA, 3)	3 (NA, 3)	1 (NA, 1)	1 (NA, 1)	8 (NA, 8)
**Not located**	**1 (0, 1)**	**9 (7, 2)**		**7 (4, 3)**	**17 (11, 6)**
Loss of VHF signal on the next day after release	1 (0, 1)	5 (5, 0)		3 (3, 0)	9 (8, 1)
Failure of migration due to a typhoon (found downriver from release point)		3 (1, 2)			3 (1, 2)
Presumed dead soon after release (found downriver from release point)		1 (1, 0)		1 (1, 0)	2 (2, 0)
Tag retrieval downriver of release point				3 (NA, 3)	3 (NA, 3)

#### Fate of tracked salmon

Eighteen of the 48 actively tracked salmon were confirmed to reach spawning tributaries or sites in the main river and were categorized as ‘located at spawning site’ (Table 3, Fig. 2 and Fig. 3). Of these, 15 were confirmed to have arrived at the spawning site, 2 were recaptured in a tributary, and 1 tag package was retrieved at a tributary (Table 3). Amongst the 18 fish, 8 settled in natural spawning habitats (main channel or non-enhanced tributaries, Fig. 3a) and 10 settled in enhanced tributaries.

Thirteen salmon were not tracked to their spawning site, classified as ‘censoring of tracking’, because of recapture in the main channel ($n=1$), the release of tag packages before arrival ($n=9$) and loss of the VHF signal after several days of tracking, including difficulties in tracking their entire upriver migration ($n=3$) (Table 3). The salmon with the longest successive tracking distance was included in this category. This salmon was tracked 93 km upstream of the release point over 6 days (Fig. 3a and c). The whereabouts after this point remained unknown, but the carcass was found in the middle basin 30 days after its last location. Another salmon carcass, tracked up to 41 km from the release point, was also found flowing from upriver in the middle basin (70 km upriver from release point, X symbol in Fig. 3a) after 21 days from last location (23 days from release). Both salmon were released in October.

The remaining 17 salmon were not confirmed to migrate upriver. This was due to the loss of the signal on the day after release ($n=9$), failure to migrate upriver due to a typhoon in October 2017 ($n=3$), presumed death shortly after release ($n=2$) and retrieval of tag packages near or downriver of the release point ($n=3$) (Table 3).

Most salmon, including those categorized as ‘not located’, moved away from around the release point within 24 h, although prolonged staying behaviour was observed in eight individuals (October: $n=7$, November: $n=1$). Of the eight, five resumed migration within 3 days. Two of these five were affected by rising waters caused by the typhoon in October 2017 and resumed migration once the water subsided. The remaining three individuals stayed >4 days, with durations of 4 ($n=1$), 5 ($n=1$) and 8 days ($n=1$).

#### Differences in migration distance and duration between months

The spawning sites of chum salmon returning in October were mainly located in the middle basin of the Kitakami River system, whereas those returning in November were located in sites from the narrow section to the lower basin (Fig. 3a). The duration and distance to spawning significantly differed between chum salmon migrating in October and November (Log-rank test, ‘time-to-event’ model: ${\chi}^2=9.14$, $P<0.01$; ‘distance-to-event’ model: ${\chi}^2=9.05$, $P<0.01$). The Kaplan–Meier curves estimated that the median duration and distance to the spawning site of salmon returning in October were 6 days (95% confidence interval (CI): 5–NA days) and 67 km (95% CI: 52–NA km) from release, whereas those returning in November were 3 days (95% CI: 2–NA days) and 47 km (95% CI: 13–NA km) from release (Fig. 3b and d). The upper bound for the 95% CI in each model could not be determined due to the limited sample size, which arose from the difficulties associated with tracking their entire upriver migration. The distance of the last point correlated with the elapsed days after release, and the segment regression model showed lower AIC and BIC values than the simple linear model (Supplementary Table S4). The validity of setting break point was confirmed using the Davies test ($P<0.01$). The larger regression slope was 16.9 $\mathrm{km}\cdotp{\mathrm{day}}^{-1}$ (95% CI: 13.4–20.3 $\mathrm{km}\cdotp{\mathrm{day}}^{-1}$) and the smaller regression slope was 9.3 $\mathrm{km}\cdotp{\mathrm{day}}^{-1}$ (95% CI: 13.4–20.3 $\mathrm{km}\cdotp{\mathrm{day}}^{-1}$) (Fig. 3c, Table 4).

#### Migratory speed

**Figure 4:**
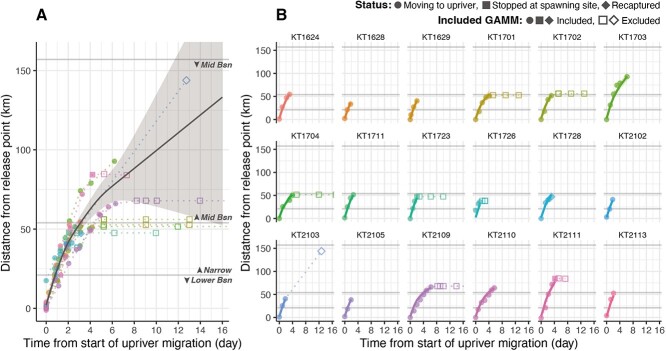
Migratory speed of chum salmon migrating upriver in the Kitakami River. The relationship between the number of days after starting the upriver movement and migration distance were modelled by GAMM. The fixed effects were shown in (A), and fitting result for each salmon (i.e. including random effect) was shown in (B). The upstream migration path of each salmon is represented by each group of coloured points connected by a dotted line. The shape of each point indicates the movement status of salmon at the indicated locations (circles: moving upriver; rectangles: stopped at spawning site; diamonds: recaptured by local fishermen). The filled symbols represent the data used for the GAMM, whilst the open symbols represent the data not used for the GAMM. The solid lines and shaded areas depict the predicted lines and the 95% CIs of the GAMM. Horizontal lines denote the thresholds of the three sections in the Kitakami River (Lower Bsn: lower basin; Narrow: narrow section; Mid Bsn: middle basin).

To estimate the daily migratory speed of salmon, the relationship between migration distance and time from the start of upriver migration was modelled by GAMM using successful tracking data (Fig. 4, $n=18$). The GAMM, which included the random intercept and slope for individual, had the lowest AIC and BIC (Supplementary Table S2). For the autocorrelation structure, the GAMMs with AR1 and AR2 correlation structures showed the lowest BIC and AIC (Supplementary Table S2), respectively, but the difference in AIC between the two models was small ($\mathrm{AI}{\mathrm{C}}_{\mathrm{AR}1\;\mathrm{model}}-\mathrm{AI}{\mathrm{C}}_{\mathrm{AR}2\;\mathrm{model}}=0.26$; $\mathrm{BI}{\mathrm{C}}_{\mathrm{AR}1\;\mathrm{model}}-\mathrm{BI}{\mathrm{C}}_{\mathrm{AR}2\;\mathrm{model}}=-4.27$); therefore, the GAMM with AR1 correlation structure was determined. Finally, the GAMM, which included fixed effects for a smoothed spline function and intercept, the random intercept and slope for individual and the AR1 correlation structure, showed the lowest AIC and BIC (Table 5, Supplementary Table S5, Supplementary Fig. S5).

The estimated times to reach the narrow section and the middle basin were 1 and 3 days, respectively, from the start of upriver migration (Fig. 4a). GAMM predictions indicated that migration speeds decreased as chum salmon moved upriver (Fig. 4). The 95% CI area for the GAMM expanded after 7 days because of the lack of samples beyond this point (Fig. 4, Supplementary Fig. S5). The migration speed varied between individuals (Fig. 4b), and the salmon with longer migration distances (e.g. KT1703 and KT2111 in Fig. 4b) tended to migrate upriver more quickly than those with shorter migration distances (e.g. KT1701, KT1702, KT2109 and KT2110 in Fig. 4b).

**Table 4:** Parameter estimates from the segmented regression model, including standard errors and 95% CIs for the two slopes and the break point

**Parameter**	**Estimates**	**Std. Error**	**95%CI**
*Slope1*	15.53	2.50	10.20–20.87
*Slope2*	5.52	1.77	1.75–9.29
*Break point*	2.84	0.85	1.02–4.66

**Table 5:** Parameter estimates for the GAMM. The fixed, random effects and autocorrelation coefficient were summarized. Standard errors, 95% CIs, estimated degrees of freedom (edf) and reference degrees of freedom (Ref.df) are included

**Parameter**	**Type of effect**	**Estimates**	**SE**	**95% CI**	**edf**	**Ref.df**
*interercept*	Fixed	32.75	1.37	30.06–35.44		
*s*(*t*)	Fixed				4.29	4.29
*s*(*ID*, bs = ‘re’)	Random				0.00	17.00
*s*(*t*, *ID*, bs = ‘re’)	Random				9.51	17.00
$\phi$	Correlation	0.56				

**Figure 5:**
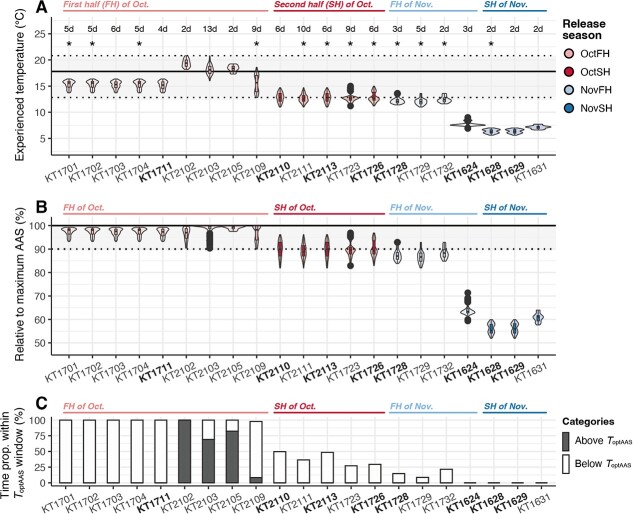
Estimation of experience temperature and thermal performance of AAS in Kitakami River chum salmon during upriver migration. The horizontal lines in (A) and (B) indicate the optimal temperature (bold line, ${T}_{\mathrm{optAAS}}$) and optimal temperature window for the absolute aerobic scope (dotted lines, defined by 90% of maximum value of AAS). The experience temperatures of individuals with bold IDs were inferred from data logger-recorded temperature, whereas those of others were inferred from river temperature. The plots were sorted by release date. (A) Thermal occupancy of chum salmon, represented with violin and box plots. The ends of each violin and box plot represent the minimum and maximum values. The maximum lengths of the ends are constrained to 1.5 times the interquartile range from the first and third quartiles, respectively, with values outside the min–max ranges shown as outliers. Filled colours denote release season. The value and asterisk above each plot represent the tracking duration (days) and the tracking result, respectively, of the individual for which spawning sites were located (no asterisk indicates that the tracking of the individual was censored). (B) Estimates of chum salmon’s AAS relative to the maximum AAS. (C) Estimates of the proportion of time spent within ${T}_{\mathrm{optAAS}}$ windows for each salmon. Filled colours denote the time proportions above ${T}_{\mathrm{optAAS}}$ (dark colour) and below ${T}_{\mathrm{optAAS}}$ (light colour).

## Thermal Occupancy and Performance of Migrating Salmon

### Experienced temperature and aerobic performance of tracked salmon

Based on the temperature records from the data loggers (October: $n=4$, November: $n=4$) and river temperature data (October: $n=10$, November: $n=3$), the experienced temperatures of the salmon were estimated. The temperatures experienced by the salmon released in October and November ranged from 11.0 to 20.8°C and 5.6 to 13.6°C, respectively (Fig. 5a). Comparing the experienced temperature with the thermal profiles of AAS estimated from early October-run fish in the previous study (Fig. 1c, Supplementary Table S3) (Abe *et al.,* 2019), all salmon in the first half of October migrate upriver within ${T}_{\mathrm{optAAS}}$ window (KT1701–KT2109 in Fig. 5b, c). The proportion of time spent on the high and low sides of the ${T}_{\mathrm{optAAS}}$ window differed for salmon released in 2017 and 2021. For salmon released in early October 2021, the high side of the ${T}_{\mathrm{optAAS}}$ window accounted for more than half of the time proportion of the salmon released in early October 2021, except for one fish (KT2109 released on 12 October), whereas no salmon experienced the temperature above ${T}_{\mathrm{optAAS}}$ (Fig. 5b, c). Salmon released in the second half of October spent less than half of their time within their ${T}_{\mathrm{optAAS}}$ window, but their AAS did not fall <80% of the AAS maximum (Fig. 5b, c). In contrast, for salmon migrating in November, the time occupancy within the ${T}_{\mathrm{optAAS}}$ window was <25% for the salmon in the first half of the month, and those migrating in the second half of the month did not experience the temperature with the ${T}_{\mathrm{optAAS}}$ window (Fig. 5b, c).

### Thermal performance estimation of Kitakami River chum salmon migrating upriver

**Figure 6:**
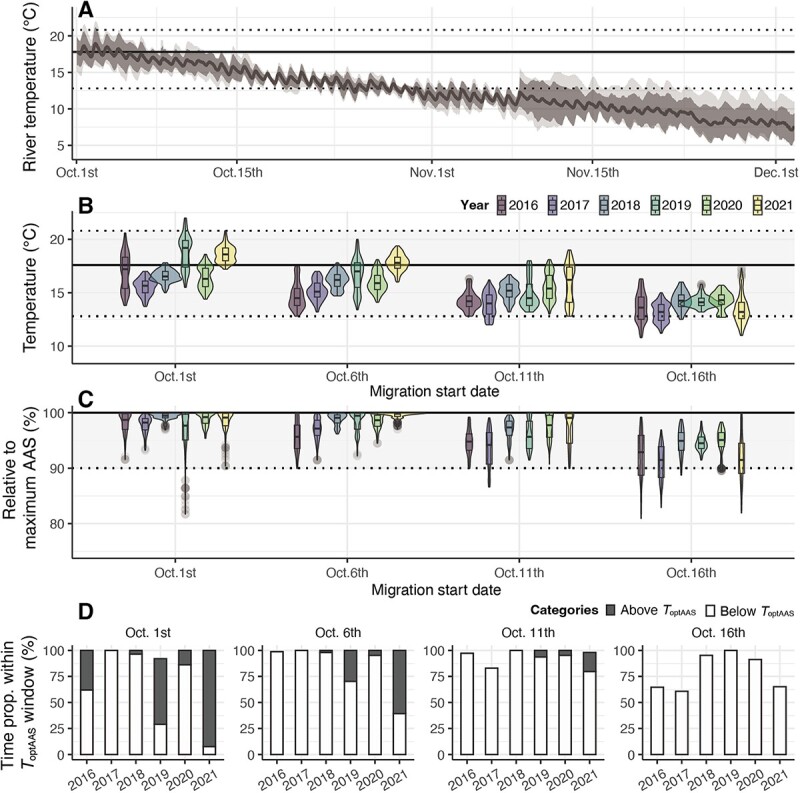
Estimation of water temperatures experienced by chum salmon in the Kitakami River during upriver migration. Horizontal lines in (A–C) represent the optimal temperature for the absolute aerobic scope (black bold line, ${T}_{\mathrm{optAAS}}$) and the optimal temperature window for the absolute aerobic scope (dotted lines, ${T}_{\mathrm{optAAS}}$ window). The ${T}_{\mathrm{optAAS}}$ window was defined as the temperature range within 90–100% of the maximum value of AAS. (A) Hourly means of river temperature in 2016–21. The bold grey line represents the mean river temperature, and the shaded areas indicate the SD (dark colour) and maximum and minimum (light colour) temperatures for 2016–21. (B) Estimates of chum salmon’s experienced temperature in 2016–21. The violin and box plots indicate estimates of salmon’s experienced temperatures from the release point until reaching the spawning site 100 km upstream (a 10-day journey) from each date (1st, 6th, 11th and 16th Oct.) of each year. The ends of each violin and box plot represent the minimum and maximum values. The maximum lengths of the ends are constrained to 1.5 times the interquartile range from the first and third quartiles, respectively, with values outside the min–max ranges shown as outliers. (C) Estimates of chum salmon’s AAS relative to the maximum AAS. Filled colours in Fig. B and C correspond to each year. (D) Estimates of the proportion of time spent within ${T}_{\mathrm{optAAS}}$ windows for each start date of upriver migration. Filled colours denote the time proportions above ${T}_{\mathrm{optAAS}}$ (dark colour) and below ${T}_{\mathrm{optAAS}}$ (light colour).

In October and November, the Kitakami river temperature gradually declined, with water temperatures fluctuating from year to year (Fig. 6a). In order to examine these inter-annual temperature fluctuations, the water temperatures experienced by migrating salmon were analysed using the river temperature records from 2016 to 2021 (Fig. 6a). Based on the 6-year record, the water temperature in the first week of October typically started within the ${T}_{\mathrm{optAAS}}$ window, where the ${T}_{\mathrm{optAAS}}$ was approximately at centre of the daily temperature fluctuations (Fig. 6a). Towards mid-October, river temperatures began shifting away from ${T}_{\mathrm{optAAS}}$, eventually moving out of the ${T}_{\mathrm{optAAS}}$ windows around 1 November (Fig. 6a). After November, the river temperature did not rise above the lower boundary of ${T}_{\mathrm{optAAS}}$ window (Fig. 6a).

We focused on salmon that began migrating in the first half of October, when the AAS of chum salmon was estimated (Abe *et al.,* 2019,), and examined the water temperatures they experienced during migration. Based on the GAMM-predicted migration speed (Fig. 4a), the migration duration from the lower basin (release point) to 100 km upriver was estimated to take 10 days, and the experienced temperatures were estimated for 10 days from the date of departure from the release point. As expected from comparing the thermal AAS profile with 6-year river temperature records, salmon that began migration on 1 October spent almost all of their migration within the ${T}_{\mathrm{optAAS}}$ window (Fig. 6b–d), and the low-temperature portion of the ${T}_{\mathrm{optAAS}}$ window accounted for more than half of the time, except in 2019 and 2021 (Fig. 6b–d). For salmon that departed on 6 and 11 October, the ${T}_{\mathrm{optAAS}}$ window still accounted for the majority of the time proportion in any cases, and the time proportion within the lower portion of ${T}_{\mathrm{optAAS}}$ window increased (Fig. 6b–d). However, for salmon departing on 16 October, none were expected to experience temperatures higher than ${T}_{\mathrm{optAAS}}$, and the proportion of time within the ${T}_{\mathrm{optAAS}}$ window was expected to decrease compared to fish departing earlier, falling <75% of AAS maxima in 3 years (2016, 2019 and 2021; Fig. 6b–d).

## Discussion

To date, detailed studies have been conducted on the ecology of upriver migration and thermal performance, along with their interrelationships, in Pacific salmonids (Clark *et al.,* 2011; Eliason *et al.,* 2011; Eliason *et al.,* 2013; Raby *et al.,* 2016). However, investigations into the duration of the chum salmon run have been sparse, with only one study reporting that adults reach their spawning site within a few days in a short river on the Sanriku coast (Nobata *et al.,* 2022). Additionally, regarding the thermal performance of AAS, measurements have been conducted only on two populations (Abe *et al.,* 2019). One of these populations was Kitakami River chum salmon, and the present study provides information about the upriver movement from the lower basin to the spawning grounds of the chum, as well as their ecological characteristics.

In this study, some salmon exhibited staying behaviour shortly after release. Although most individuals started their upriver migration within 24 h, eight did not resume their migration for >3 days. It is possible that the staying behaviour observed within 24 h was caused by the effect of tagging, as a previous study on chum salmon reported the anaesthesia effects lasted 1–6 h (Hayashida *et al.,* 2013). Of the eight with longer stays, two were delayed by rising waters from a typhoon in 2017, but resumed migration once the waters subsided, which took ~3 days. However, the reason for prolonged staying behaviour of the remaining of six individuals is unknown. The tagging procedure was carried out similarly to previous studies, and this method did not have a significant impact on upriver migration behaviour (Makiguchi *et al.,* 2007, 2008) or spawning behaviour (Tsuda *et al.,* 2006). We cannot entirely rule out the possibility of some tagging impact but it is considered that the tagging did not significantly interfere with their natural behaviour.

### Stock characteristics of Kitakami River chum salmon

Japanese chum salmon stocks are believed to be maintained primarily through hatchery-origin salmon. The Kitakami River also has 13 hatcheries in the tributaries of the middle basin and the narrow section, but all of the hatcheries are small-scale and the number of returning adults captured by hatcheries is low, averaging ~600 fish per year for the past decade for each hatchery of the middle basin. In contrast, in the Kitakami River system, 40 000–50 000 salmon (not used for hatchery-enhanced programs) were caught throughout the fishing season (October–December) in the lower basin. In this study, 48 salmon equipped with electronic tags were released into the lower basin of the river, and 18 salmon were tracked to their spawning grounds (Table 3). Of the 18 salmon, eight (44%) were observed in spawning habitats unrelated to hatcheries. Moreover, only three of the 53 chum salmon were recaptured by fishermen near hatchery spawning grounds (Table 3), indicating a low recapture rate (5.7%). These data suggest that wild-origin fish make a non-negligible contribution to sustaining the Kitakami River population.

### Features of the upriver migration of Kitakami River chum salmon

The estimates of migratory speed varied between 6 and 22 $\mathrm{km}\cdotp{\mathrm{day}}^{-1}$ (Fig. 4) in this study. Although the CI was wide due to the small number of individuals tracked from the middle basin onwards, the migration speed estimated by GAMM was generally consistent with the 144-km migration distance for salmon recaptured 13 days after release (KT2103, Fig. 4), supporting the validity of the GAMM. The estimated migratory speed is broadly in accordance with a previous study of chum salmon migrating upriver from the middle basin to the upper basin of a Japanese river (6.6–19.5 $\mathrm{km}\cdotp{\mathrm{day}}^{-1}$) (Aruga *et al.,* 2009). The migratory speeds are also similar to those of other salmonid species: sockeye salmon (*Oncorhynchus nerka*): 17–40 $\mathrm{km}\cdotp{\mathrm{day}}^{-1}$ (English *et al.,* 2005), Chinook salmon (*Oncorhynchus tshawytscha*): 10–30 $\mathrm{km}\cdotp{\mathrm{day}}^{-1}$ (Keefer *et al.,* 2004), coho salmon (*Oncorhynchus kisutch*): 7–21 $\mathrm{km}\cdotp{\mathrm{day}}^{-1}$ (Raby *et al.,* 2012) and Atlantic salmon: 18–27 $\mathrm{km}\cdotp{\mathrm{day}}^{-1}$(Øklamd *et al.,* 2001). Through the active tracking of Kitakami River chum salmon, it was observed that their migratory speed gradually decreased as they progressed upriver, also concurring with previous studies on salmonids (Øklamd *et al.,* 2001; Keefer *et al.,* 2004; English *et al.,* 2005), including chum salmon (Aruga *et al.,* 2009). River flow is generally considered an important environmental factor affecting migration speeds for Pacific salmon (Makiguchi *et al.,* 2007, 2008; Aruga *et al.,* 2009; Martin *et al.,* 2015). In fact, the salmon that experienced a typhoon in 2017 either failed to migrate or exhibited staying behaviour until the increased flow subsided, indicating that although extreme in this case, the river current had a considerable impact on migration speed and migration success. In fast-flowing environments, salmon migrating upriver are believed to combine swimming at above maximum sustained speed (critical swim speed) and resting, resulting in a reduction in migration speed (Makiguchi *et al.,* 2008; Martin *et al.,* 2015). The slope of the Kitakami River gradually rises after a narrow area (Table 1), so the flow speed is expected to increase. Thus, the reduction in migration speed after the lower basin could be attributed to the flow environment of the river.

Other factors affecting migration speed may include distance to spawning sites. In this study, all individuals exhibited a decrease in migration speed as they progressed upstream, but the extent of the decline and the overall migration speed varied between individuals. Although the limited sample size precludes definitive discussion, individuals with longer migration distances (e.g. KT1703 and KT2111 in Fig. 4b) tended to move upriver more quickly than those with shorter migration (e.g. KT1701, KT1702 and KT1704 in Fig. 4b). In Atlantic salmon, a ‘searching phase’ has been observed, during which they migrate upriver whilst searching for their spawning grounds (Øklamd *et al.,* 2001). Although a clear searching phase for chum salmon was not identified in this study, it is likely that individuals with shorter migration distances begin searching for their spawning sites earlier than those with longer distances to cover.

In Kitakami River chum salmon, the spawning site and duration of upriver migration differed between fish returning in October and those returning in November (Fig. 3). The salmon returning in October had a migration duration of >5 days and predominantly spawned in the main channel and tributaries in the middle basin of the Kitakami River, which is >54 km upriver from the release point (Table 1). On the other hand, the salmon returning in November had a shorter migration duration and spawned in the narrow section or lower basin, reaching their spawning sites in just a few days (Fig. 3). The observations, earlier run salmon tending to further migrate upriver, matched the chum salmon catch information in the Kitakami River (Fig. 1b) and the general tendency of Pacific salmon (Eliason *et al.,* 2011; Eliason *et al.,* 2013). However, it should be noted that the fish classified into ‘censoring of tracking’ were tracked for a longer period of time than the fish whose spawning sites were identified (Fig. 3). Therefore, it is possible that the estimated migration duration and distance for both months were underestimated. In the present study, we were not able to track salmon to the uppermost spawning area near the upriver hatchery spawning sites, 121–157 km upriver from the release point (Fig. 1a). Thirteen salmon were classified as ‘censoring of tracking’, and six of these were censored in the middle basin (Fig. 3d, Supplementary Fig. S4), implying that the middle basin provides potential spawning habitats for these fish. Furthermore, five out of the six were released in October (Supplementary Fig. S4), indicating that the extent of underestimation may be greater for October salmon than for November salmon.

Regarding the maximum duration of the upriver migration of Kitakami River chum salmon, two chum salmon carcasses were found drifting ashore from upstream 23 and 36 days after release. Considering that the VHF-equipped salmon were active on their spawning grounds for 5–13 days (Fig. 4), it is unlikely that the migration was prolonged to >1 month, as observed in other salmonid species migrating to major rivers exceeding thousands of kilometres such as Atlantic salmon (Øklamd *et al.,* 2001) and sockeye salmon (Eliason *et al.,* 2011). Indeed, the GAMM of migration speed indicated that the uppermost spawning area could be reached in ~2–3 weeks (Fig. 4a). However, this migration speed reflects only the upriver migration speed and does not account for the time spent downstream, suggesting that it may take a few more days. Six of the 48 individuals were observed to linger around the release point for several days for unknown reasons, and five of these were salmon returning in October, indicating a possible tendency for October salmon to remain downstream for a short period before moving upriver. Assuming a stay of a few days in the lower basin, the upriver migration duration of the October-returning salmon can be estimated to be in the range of 1–3 weeks, as the salmon took ~3–5 days to reach the middle basin (Fig. 4a). In contrast, the migratory duration of the November-returning salmon can be estimated to be <1 week because the salmon took <5 days to reach the end of the narrow section (Fig. 4a) and November salmon did not show the staying behaviour in downriver.

These tracking results suggest that salmon returning in October and November have different ecological characteristics. The genetic structure of the Kitakami River population is not fully understood, although a previous report implied genetic differentiation amongst tributaries (Tsukagoshi *et al.,* 2016). In general, Pacific salmonids are believed to form fine-to-large spatial and temporal genetic structures (Fillatre *et al.,* 2003; Olsen *et al.,* 2008, 2011; Kitanishi *et al.,* 2009; O’Malley *et al.,* 2010; May *et al.,* 2023). The migration timing of the Pacific salmonid is strongly influenced by genotype (O’Malley and Banks, 2008,), and temporal factors are thought to influence genetic differentiation more strongly than spatial factors (Olsen *et al.,* 2008; Yokotani *et al.,* 2009). Although it is difficult to determine the extent to which genetic isolation occurs between tributaries for salmon returning in the same month, it is likely that the ecological and physiological characteristics of salmon returning during the same time are similar because of shared migration environments. Further genetic research is necessary to specify the spatiotemporal structure of the Kitakami River chum salmon population in greater detail.

### Comparison of the AAS thermal profile with the temperature experienced during upriver migration

The Kitakami River chum salmon returning in October exhibited different migration characteristics compared to those returning in November, and notably varied in thermal occupancy during migration. Based on the temperature data, October-returning salmon were expected to maintain high aerobic performance (>80% $\mathrm{AA}{\mathrm{S}}_{\mathrm{max}}$) (Fig. 5b, c), and, in particular, those in the first half of October spent their time within the optimal temperature range of AAS (${T}_{\mathrm{optAAS}}$ window) (Fig. 5b–d). In contrast, November-returning salmon did not spend their time in the ${T}_{\mathrm{optAAS}}$ window (Fig. 5b–d), and those returning in the second half of November did not experience higher temperatures than the lower boundary of the ${T}_{\mathrm{optAAS}}$ window (Fig. 5b–d). The differences in AAS performance between October- and November-returning salmon could allow for several interpretations. First, November-returning salmon may have different AAS thermal profiles compared to October-returning salmon. Each population of Pacific salmon is thought to have a thermal performance profile for AAS that are closely related to its thermal environment (Eliason *et al.,* 2011; Eliason *et al.,* 2013). For chum salmon, it has also been found that salmon returning to the Kitakami River in October have a different thermal performance curve with high ${T}_{\mathrm{optAAS}}$ window compared to those returning to another Sanriku coastal river in December (Abe *et al.,* 2019). The November-returning Kitakami River salmon differed in habitats, both spatially (spawning site) and temporally (migration timing); therefore, it is likely that these two groups may have different AAS thermal profiles. Alternatively, November-returning chum salmon may not require high aerobic performance. Previous studies have shown that salmon populations with low migration challenges exhibit low AAS performance (Eliason *et al.,* 2013; Abe *et al.,* 2019). The migration difficulties of Kitakami River chum salmon returning in November were lower than those returning in October. In any event, the thermal performance of salmon in their natural environment in November cannot be discussed solely on the basis of the AAS thermal profile obtained from October-returning salmon, and further research is required.

Relative to a previously reported thermal performance for AAS, the chum salmon in October 2017 and 2021 migrated upriver within the ${T}_{\mathrm{optAAS}}$ window but tended to migrate below the optimal temperature for AAS (${T}_{\mathrm{optAAS}}$) (Fig. 5). Estimation of temperatures experienced by the chum salmon across several years supported our observation that the October-run salmon in this river would mainly migrate through the low-temperature range of ${T}_{\mathrm{optAAS}}$ window. Thermal performance curves of AAS are known to be generally consistent with ecologically relevant temperatures, but it has been reported that the peaks of the performance curves do not exactly match the modes or averages of the temperatures (Clark *et al.,* 2011; Norin *et al.,* 2014; Raby *et al.,* 2016). In these unmatched cases, the species typically spent most of their time below ${T}_{\mathrm{optAAS}}$ or showed preference for temperatures below ${T}_{\mathrm{optAAS}}$ (Clark *et al.,* 2011; Norin *et al.,* 2014; Raby *et al.,* 2016). For example, juvenile barramundi (*Lates calcarifer*), a eurythermal tropical fish, acclimated at 29°C showed the highest AAS ~38°C, but the juveniles preferred 31°C (Norin *et al.,* 2014). In Pacific salmonids, pink salmon and coho salmon were estimated to have higher ${T}_{\mathrm{optAAS}}$ than the ecologically relevant temperature range (Clark *et al.,* 2011; Raby *et al.,* 2016). Furthermore, the optimal temperatures in both cases were close to the upper limit of the historical river temperatures over the past 10–50 years (Clark *et al.,* 2011; Raby *et al.,* 2016).

The results of the present and previous studies could reflect several possibilities. For example, the upward trajectories of the AAS performance curve can function as a buffer for inter-year temperature fluctuations. Although there is some evidence that Pacific salmon may postpone river entry in response to adverse conditions (Goniea *et al.,* 2006; Mantua *et al.,* 2015), in most cases, salmon embark on their migratory journey towards their natal freshwater habitat on a relatively consistent schedule (Crozier *et al.,* 2008). Thus, maintaining such a thermal performance curve for AAS can have the advantage of allowing high aerobic performance even in years with high river temperatures during long-term temperature fluctuations. In fact, the Kitakami River temperature varied between years, even within 6 years, and the chum salmon are expected to maintain high AAS performance in 2019 and 2021, when the river temperatures in early October were high compared with those in other years, supporting this hypothesis (Fig. 6b–d). Alternatively, maximizing aerobic performance may not be required for Kitakami River chum salmon to accomplish their spawning migration. There is little doubt that aerobic metabolism and high aerobic performance are required for adult sockeye salmon populations with severe migration difficulties. However, chum salmon with pink and coho salmon have shorter migration distances and less challenging upriver migration than sockeye salmon. For salmon with low migration challenges, maximizing their aerobic performance may be less ecologically important than for those salmon with rigorous migration requirements (Eliason *et al.,* 2013).

The ecological challenges that Pacific salmonids face during their spawning migration include not only maintaining high aerobic performance to overcome river currents but also reducing migration costs. Pacific salmonids on their spawning migration stop feeding upon arrival at their natal coast, but then have to complete their run with limited somatic energy. From a metabolic cost perspective, chum salmon may benefit from migrating upriver within the low-temperature area of ${T}_{\mathrm{optAAS}}$. AAS are calculated from difference between maximum oxygen uptake and the maintenance cost, and the thermal performance curve forms an upward convex curve (Payne and Smith, 2017). Meanwhile, maintenance costs increase exponentially with temperature (Payne and Smith, 2017). On the low- and high-temperature sides of the thermal performance curve for the AAS, the maintenance cost is lower on the low-temperature side, even though the AAS itself remains comparable on both sides. As a result, the total cost of migration per unit distance is expected to be lower below ${T}_{\mathrm{optAAS}}$ due to reduced maintenance cost. For example, in a previous study, the resting metabolic rate (${B}_{\mathrm{RMR}}$, in $\mathrm{mg}{\mathrm{O}}_2\cdotp{\mathrm{kg}}^{-1}\cdotp{\min}^{-1}$) of Kitakami River chum salmon was modelled as an exponential curve with temperature ($T$, in °C), ${B}_{\mathrm{RMR}}=a{e}^{dT}$ ($a=0.707$, $d=0.088$, Abe *et al.* (2019)). According to the function, the maintenance cost increases by ~200% from the lower temperature boundary of ${T}_{\mathrm{optAAS}}$ window (12.8°C) to the higher temperature boundary (20.8°C). The absolute increases can be calculated as 45.3 $\mathrm{kJ}\cdotp{\mathrm{kg}}^{-1}\cdotp{\mathrm{day}}^{-1}$, assuming an oxycaloric equivalent of 14.1 $\mathrm{J}\cdotp \mathrm{mg}{{\mathrm{O}}_2}^{-1}$ (Videler and Videler, 1993).

The AAS is a functional metric for physiological traits, including growth rate and digestion speed. Indeed, a study on brown trout (*Salmo trutta*) showed that AAS was linked to the speed of digestion (Auer *et al.,* 2015a). However, in common killifish (*Fundulus heteroclitus*) (Healy and Schulte, 2012) and barramundi (Katersky and Carter, 2007; Bermudes *et al.,* 2010), it has been reported that the temperatures maximizing AAS do not facilitate the growth rate but rather decrease growth and sometimes lead to a reduction in body weight; increased maintenance cost was proposed as one of the explanations for this observation (Schulte, 2015). Maintaining high AAS is a cost in itself, and the optimal relationship between AAS and maintenance costs for growth depends on the environment (Auer *et al.,* 2015b). This can be seen as an analogy for spawning migrations of Pacific salmonids as well, and there seems to be an optimal AAS value (and the combination for maintenance cost) depending on the species- and population-specific context.

### Impact of elevated river temperature on Kitakami River chum salmon

Global warming is progressing, and Representative Concentration Pathways predict that average air temperatures could increase by 2–4°C by 2100. Assuming a 2 or 4°C increase from the 2016–21 river temperatures, in both cases, salmon returning in early October are most likely to encounter water temperatures exceeding the ${T}_{\mathrm{optAAS}}$ window (Fig. 6, Supplementary Fig. S6), raising concerns about the negative impacts on aerobic performance. Additionally, the proportion of time spent by salmon in the high-temperature portion of the ${T}_{\mathrm{optAAS}}$ window is also anticipated to increase significantly (Supplementary Fig. S6b, d). In addition to this, the maintenance costs are also expected to increase by 20–40% as water temperatures rise by 2–4°C. However, the maintenance costs associated with environmental temperature fluctuations are complex because recent studies have shown that the metabolic rates of fish are thermally compensated by chronic exposure to warming temperatures (Sandblom *et al.,* 2016; Gonzàlez-Ferreras *et al.,* 2023).

In addition to potential negative impacts on aerobic performance and somatic energy, studies on sockeye salmon have also suggested that a combination of factors, such as parasitic infection, stress and reduced osmotic regulation, can contribute to the en route mortality of Pacific salmon at elevated river temperatures (Farrell *et al.,* 2008; Hinch *et al.,* 2008). Although this study observed little en route mortality for salmon returning in October, rising river temperatures caused by global warming may pose a future challenge to the migratory success of chum salmon in the Kitakami River in the future. Therefore, further studies are required to assess the impact of rising temperature on the success of upriver migration and reproduction in Kitakami River chum salmon.

## Conclusions and Perspectives

Through active tracking of Kitakami River chum salmon during the spawning season, we revealed features of migration ecology in this population. Migration duration and distance varied between salmon returning in October and November, with earlier returning salmon tending to migrate towards upriver spawning sites, resulting in longer migration durations. Based on the tracking results, we estimated the thermal occupancy of Kitakami River chum salmon, which showed that October-returning salmon maintain high aerobic performance due to their spending more time within the ${T}_{\mathrm{optAAS}}$ window. In contrast, the thermal occupancy of November-returning salmon was not consistent with the AAS thermal profile inferred from the October-returning salmon in a previous study. Considering the differences in migration ecology between October- and November-returning salmon, this suggests that the November-returning salmon either have a different AAS thermal profile or do not rely on high aerobic performance. Our findings provide essential information for assessing the resilience of this population in a changing environment, offering insights into the migration ecology of chum salmon and their connection to aerobic thermal performance in the natural environment.

## Supplementary Material

Web_Material_coae087

## Data Availability

The datasets used and/or analysed in the current study are available from the corresponding author upon reasonable request.
