# Supplementary material for: Ecological features of upriver migration in Kitakami River chum salmon and their connection to aerobic thermal performance
Source: Conserv Physiol. 2024 Dec 26;12(1):coae087. doi: 10.1093/conphys/coae087 (PMC11669486; doi:10.1093/conphys/coae087)
Supplement: Web_Material_coae087 [file web_material_coae087.pdf]

## Supplemental Tables

**Supplementary Table 1:** The AIC and BIC values of Cox models.

**Supplementary Table 2:** AIC and BIC values of generalised additive mixed models (GAMMs) with different random effects or correlation structures in the relationship between distance from release site and the time from start of upriver migration.

**Supplementary Table 3:** Parameters of thermal performance curve for absolute aerobic scope

**Supplementary Table 4:** Relationship between migration distance ($D$) and time ($t$) modelled with linear model with/without segmented regression.

**Supplementary Table 5:** AIC and BIC values of GAMMs with different fixed effects in the relationship between distance from release site and the time from start of upriver migration.

Supplementary Table 1: AIC and BIC values of Cox models using time ($h(t)$) and distance ($h(D)$) to reach spawning site as hazard functions. Covariates included both timing and biological characteristics: days from October 1st (days, numeric: the number of days between the release date and Oct. 1st), half of month (hom, factor: first/second half of October/November), month (month, factor: October/November), year (year, factor: 2016/2017/2021), sex (sex, factor: male/female), and fork length (FL, numeric: fork length).

| Hazard function | Model | Covariate | df | AIC | BIC |
|---|---|---|---|---|---|
| *h*(*t*) | **COX-time-day** | ***days*** | **1** | **85.65** | **86.54** |
| | COX-time-month | *month* | 1 | 85.96 | 86.85 |
| | COX-time-hom | *half-of-month* | 3 | 88.80 | 92.47 |
| | COX-time-y | *year* | 2 | 89.14 | 90.92 |
| | COX-time-null | none | 0 | 91.85 | 91.85 |
| | COX-time-sex | *Sex* | 1 | 92.00 | 92.89 |
| | COX-time-fl | *FL* | 1 | 93.73 | 94.62 |
| *h*(*D*) | **COX-dist-day** | ***days*** | **1** | **88.24** | **89.18** |
| | COX-dist-month | *month* | 1 | 90.69 | 91.63 |
| | COX-dist-hom | *half-of-month* | 3 | 92.59 | 95.42 |
| | COX-dist-year | *year* | 2 | 92.77 | 94.66 |
| | COX-dist-null | none | 0 | 96.45 | 96.45 |
| | COX-dist-sex | *Sex* | 1 | 96.69 | 97.64 |
| | COX-dist-fl | *FL* | 1 | 97.95 | 98.89 |

Supplementary Table 2: The AIC and BIC values of generalized additive models (GAMMs). All models were estimated by restricted maximum likelihood. The best-fitting models with random effects and random effects/correlation structure are indicated in bold.

| **Model** | **Formula** | **Random** | **Corr. Str.** | **df** | **AIC** | **BIC** |
|---|---|---|---|---|---|---|
| GAM0 | *D* ~ *s*(*t*) + *intercept* | | | 4 | 552.33 | 561.60 |
| GAMM-id1 | *D* ~ *s*(*t*) + *intercept* | *intercept* \| *ID* | | 5 | 526.01 | 537.60 |
| GAMM-id2 | *D* ~ *s*(*t*) + *intercept* | *t* \| *ID* | | 4 | 552.33 | 561.60 |
| GAMM-id3 | *D* ~ *s*(*t*) + *intercept* | *intercept* + *t* \| *ID* | | 7 | 499.94 | 516.17 |
| GAMM-id4 | *D* ~ *s*(*t*) + *intercept* | *s*(*ID*, bs = "re") | | 5 | 526.01 | 537.60 |
| GAMM-id5 | *D* ~ *s*(*t*) + *intercept* | *s*(*t*, *ID*, bs = "re") | | 5 | 502.78 | 514.37 |
| **GAMM-id6** | ***D* ~ *s*(*t*) + *intercept*** | ***s*(*ID*, bs = "re") + *s*(*t*, *ID*, bs = "re")** | | **6** | **497.94** | **511.85** |
| GAMM-year1 | *D* ~ *s*(*t*) + *intercept* | *s*(*year*, bs = "re") + *s*(*t*, *year*, bs = "re") | | 5 | 552.54 | 564.13 |
| GAMM-month1 | *D* ~ *s*(*t*) + *intercept* | *s*(*month*, bs = "re") + *s*(*t*, *month*, bs = "re") | | 5 | 554.33 | 565.91 |
| GAMM-hom1 | *D* ~ *s*(*t*) + *intercept* | *s*(*hom*, bs = "re") + *s*(*t*, *hom*, bs = "re") | | 5 | 554.33 | 565.91 |
| **GAMM-id6-AR1** | ***D* ~ *s*(*t*) + *intercept*** | ***s*(*ID*, bs = "re") + *s*(*t*, *ID*, bs = "re")** | **AR1** | **7** | **488.73** | **504.95** |
| GAMM-id6-AR2 | *D* ~ *s*(*t*) + *intercept* | *s*(*ID*, bs = "re") + *s*(*t*, *ID*, bs = "re") | AR2 | 8 | 488.47 | 507.01 |
| GAMM-id6-AR3 | *D* ~ *s*(*t*) + *intercept* | *s*(*ID*, bs = "re") + *s*(*t*, *ID*, bs = "re") | AR3 | 9 | 490.42 | 511.28 |

Supplementary Table 3: Parameters for the two-part thermal performance curve of absolute aerobic scope (AAS). The values in parentheses represent standard error.

| Symbol | Estimates | Description |
|---|---|---|
| $S$ | 10.74 (±0.45) | Scalar equal to the maximum of absolute aerobic scope (AAS) |
| $T_{\mathrm{optAAS}}$ | 17.64 (±1.12) | Optimal temperature for AAS |
| $T_{\mathrm{crit,max}}$ | 27.84 (±0.27) | Critical temperature maxima |
| $\sigma$ | 7.43 (±3.16) | Standard deviation for the normally distributed half of the curve |

Supplementary Table 4: Relationship between migration distance (*D*) and time (*t*) modelled with linear model with/without segmented regression. The best-fitting models are indicated in bold.

| **Model** | **Formula** | **Num. of breakpoint** | **df** | **AIC** | **BIC** |
|---|---|---|---|---|---|
| Null model | *D ~ intercept* | - | 2 | 163.42 | 165.20 |
| LM1 | *D ~ t* | 0 | 2 | 144.78 | 146.56 |
| LM2 | *D ~ t + intercept* | 0 | 3 | 140.95 | 143.62 |
| **LM1-seg** | *D ~ t* | **1** | **4** | **137.57** | **141.14** |
| LM2-seg | *D ~ t + intercept* | 1 | 5 | 139.08 | 143.53 |

Supplementary Table 5: Relationship between migration distance and time spent modelled using a generalised additive mixed model (GAMM). The best-fitting models are indicated in bold. Random effect was modelled as the intercept and slope, and correlation structure was modelled with AR1. All models were estimated by maximum likelihood.

| **Model** | **Formula** | **Random** | **Corr. Str.** | **df** | **AIC** | **BIC** |
|---|---|---|---|---|---|---|
| GAMM0 | *D ~ intercept* | *s*(*ID*, bs = "re") + *s*(*t*, *ID*, bs = "re") | AR1 | 5 | 584.37 | 596.09 |
| GAMM1 | *D ~ s*(*t*) | *s*(*ID*, bs = "re") + *s*(*t*, *ID*, bs = "re") | AR1 | 6 | 566.40 | 580.47 |
| **GAMM2** | *D ~ s*(*t*) + *intercept* | ***s*(*ID*, bs = "re") + *s*(*t*, *ID*, bs = "re")** | **AR1** | **7** | **495.46** | **511.87** |

## Supplemental Figures

**Supplementary Figure 1:** Movement patterns of chum salmon after the onset of upriver migration, shown as distance traveled from the release site as a function of time (days) since the start of upriver migration.

**Supplementary Figure 2:** Fitting results of the GAMM with random effects (GAMM-id6, in Supplementary Table 2) for individual chum salmon migration.

**Supplementary Figure 3:** Temperature records from data-loggers attached to chum salmon during their migration.

**Supplementary Figure 4:** Kaplan-Meier survival curves showing the probability of chum salmon reaching upstream locations after release.

**Supplementary Figure 5:** Partial effects of smooth terms from the final GAMM model ("GAMM2" in Table S6).

**Supplementary Figure 6:** Estimates of the water temperatures experienced by Kitakami River chum salmon during their upriver migration under elevated temperature conditions and the proportion of time the salmon spent within the optimal temperature window for absolute aerobic scope ($T_{\mathrm{optAAS}}$ window) at that time.

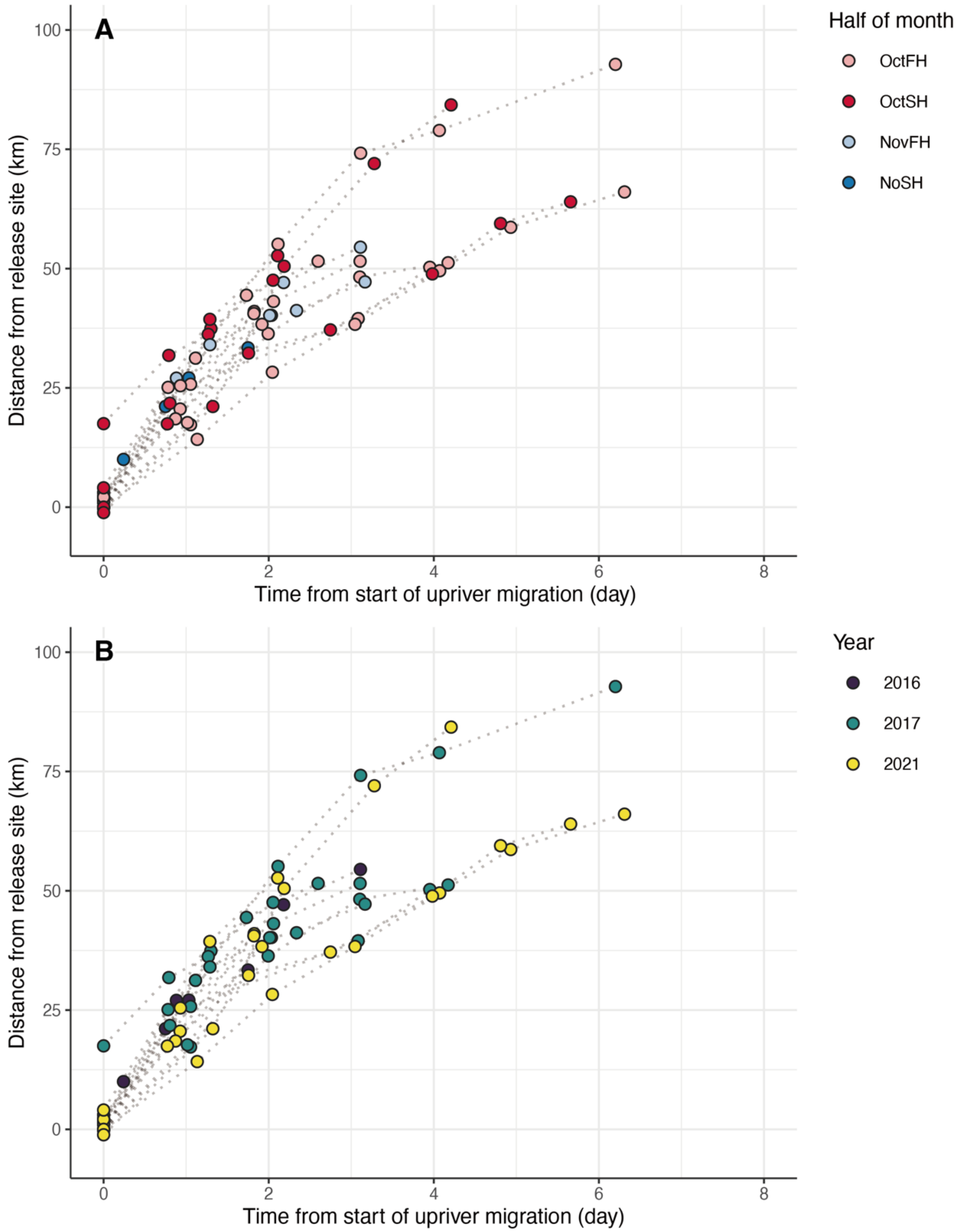


Supplementary Figure 1: Movement patterns of chum salmon after release, shown as distance traveled (km) from the release site as a function of time (days) since the start of upriver migration. Each group of points connected by a dotted line indicates a salmon trajectory of migrating upriver. (A) The data points are categorised by half-month periods: first half of October (OctyFH, light pink), second half of October (OcfSH, dark red), first half of November (NovFH, light blue), and second half of November (NovSH, dark blue). Each group exhibits distinct migration dynamics over time. (B) The data points are grouped by year: 2016 (dark blue), 2017 (dark green), and 2021 (yellow).

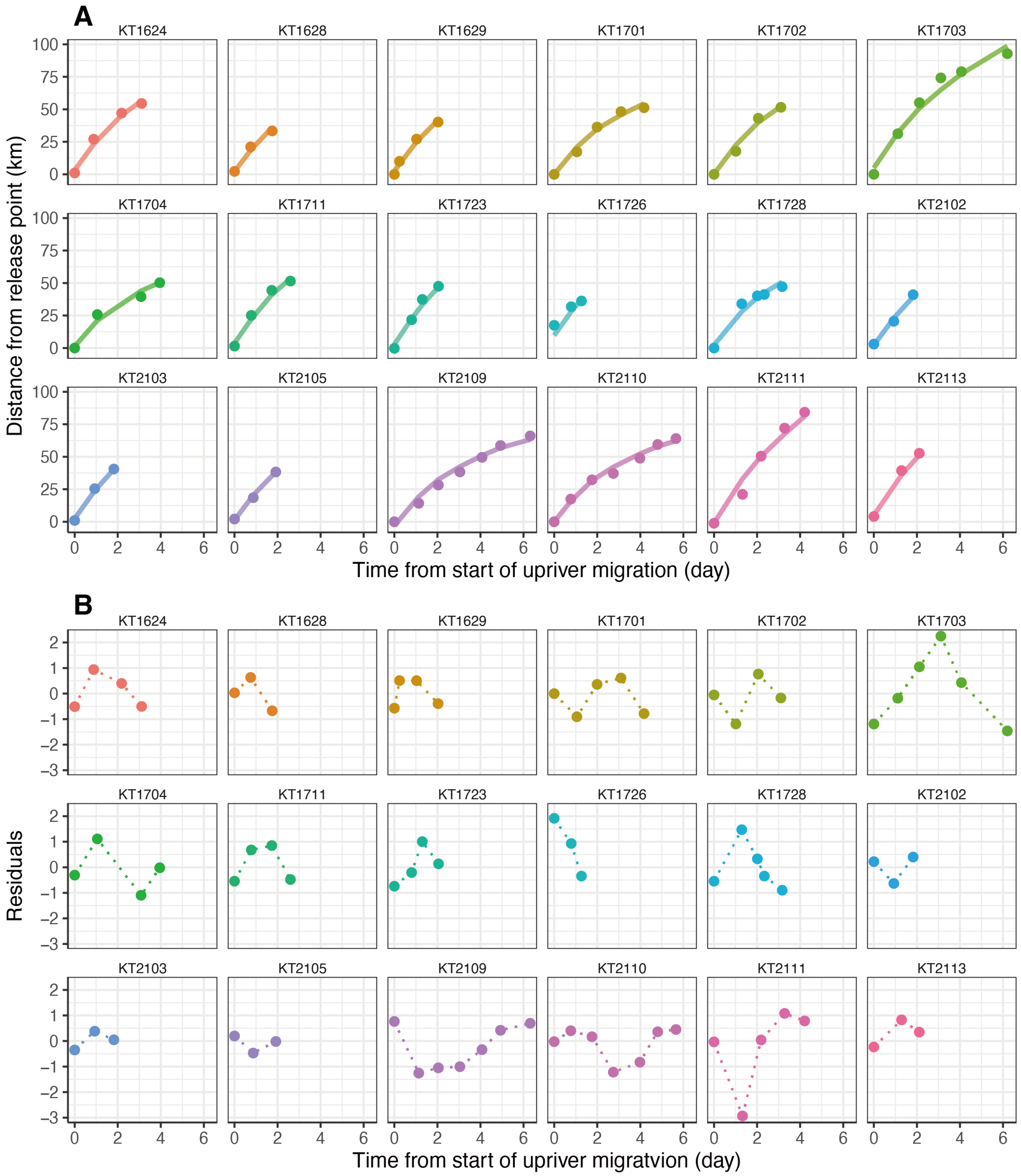


Supplementary Figure 2: Fitting results of the generalised additive mixed model (GAMM) with random effects (GAMM-id6 in Supplementary Table 2) for individual chum salmon migration. (A) Distance from the release point over time for each individual salmon, modelled using a GAMM. Each panel represents one individual, with time since the start of upriver migration on the x-axis and the distance from the release point (in km) on the y-axis. (B) Residuals of the GAMM fit, showing the difference between the observed and predicted values for each individual. Each coloured point represents a different salmon.

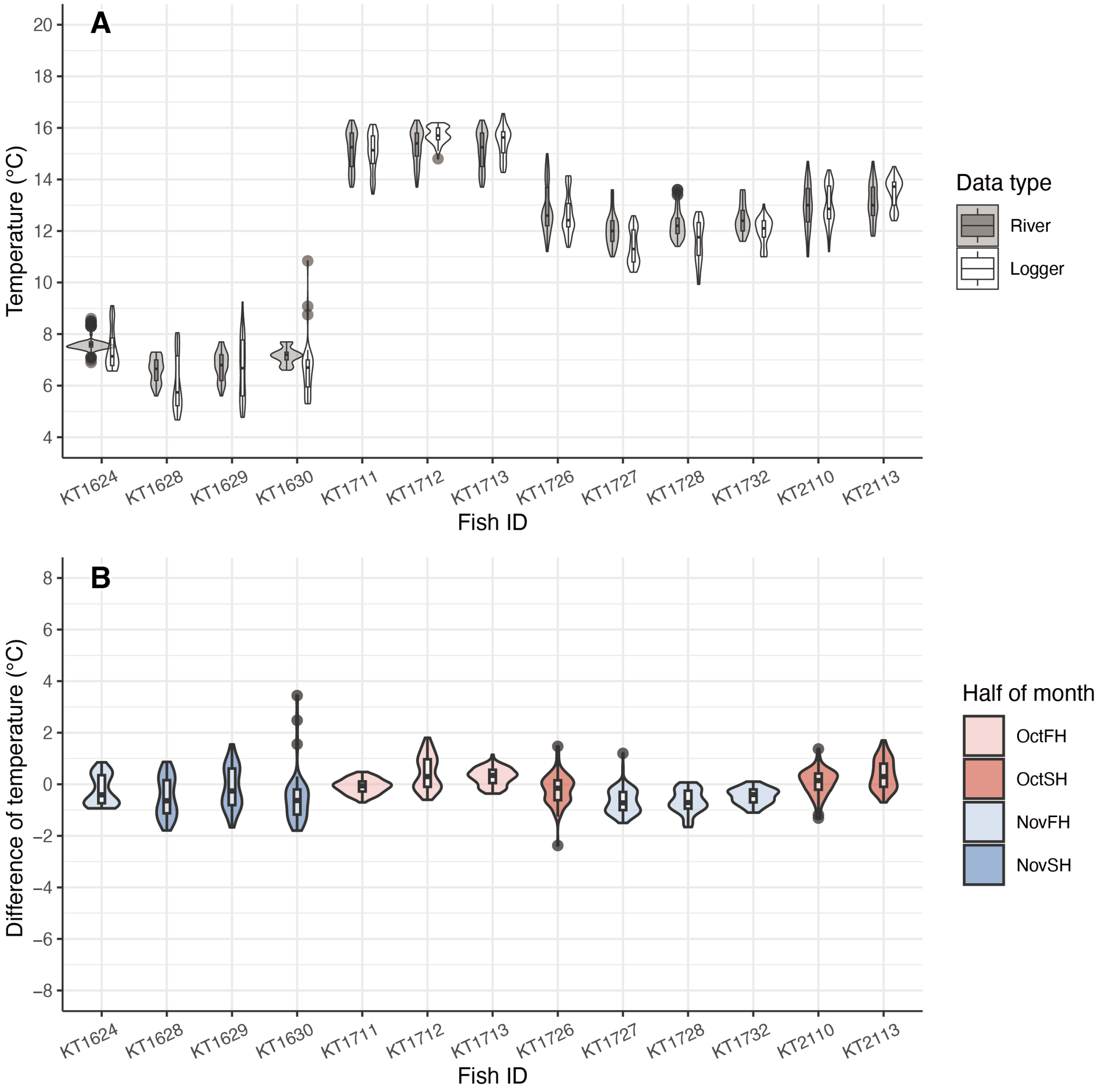


Supplementary Figure 3: Temperature records from data-loggers attached to chum salmon during upriver migration and comparison with river temperature. (A) Data-logger recorded water temperatures for each fish ID and river temperature at that time. (B) Differences between the temperatures recorded by the loggers and the ambient river water temperatures. Fish were released in either the first or second half of October or November, indicated by the colour of the violin and box plots (pink/red for October, light blue/dark blue for November).

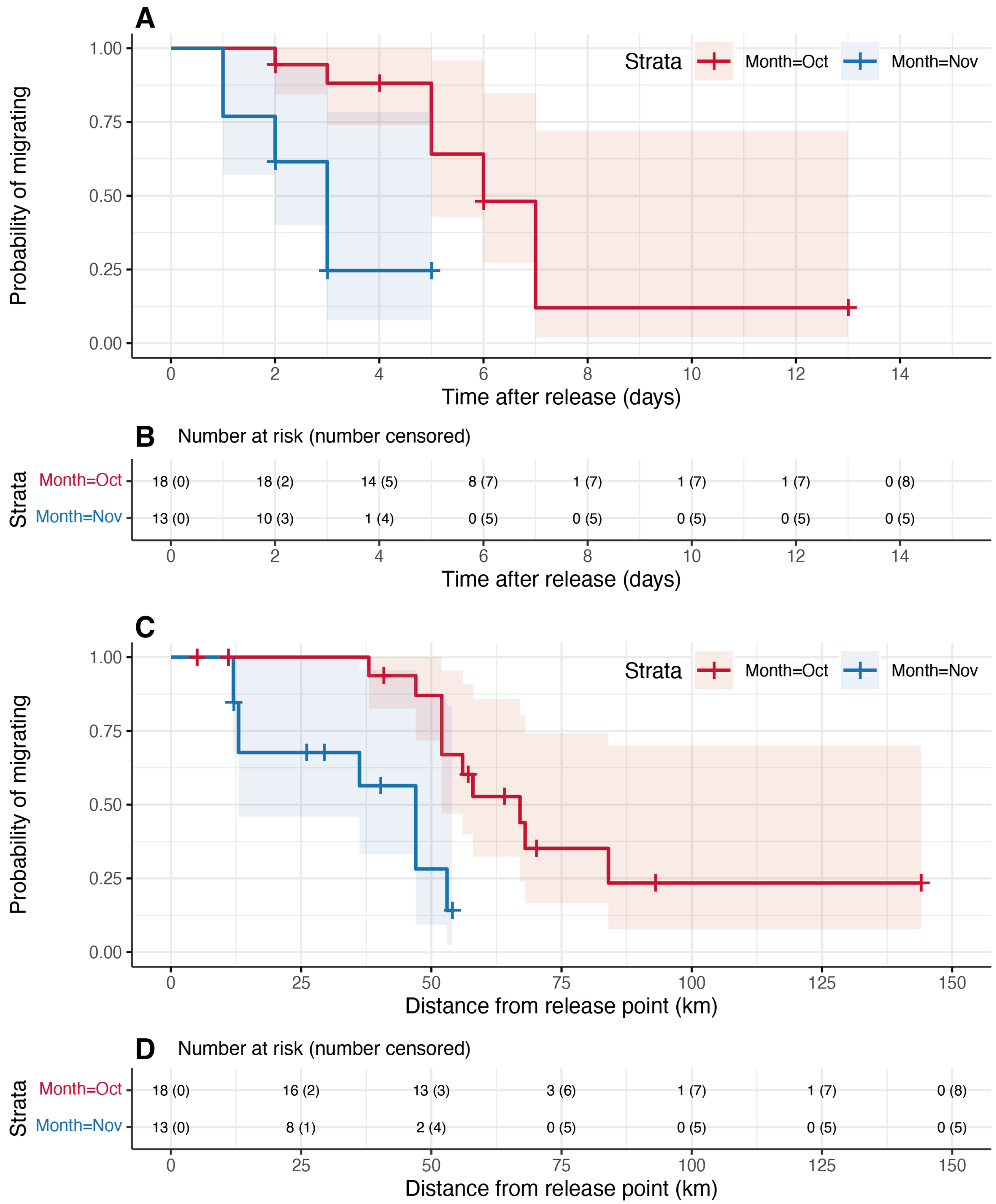


Supplementary Figure 4: Kaplan-Meier survival curves (KM curves) showing the probability of chum salmon reaching upstream locations after release. (A) KM curves of the probability of arrival as a function of time (days) after release, categorised by month: October (red) and November (blue). The shaded areas represent the 95% confidence intervals (95%CIs), and the number of fish at moving upriver (and censored) at each time point is shown in panel (B). (C) KM curves of the probability of arrival as a function of distance (km) from the release point, also stratified by month. The 95%CIs are shaded, with the number of fish moving upriver (and censored) at each time point displayed in panel (D).

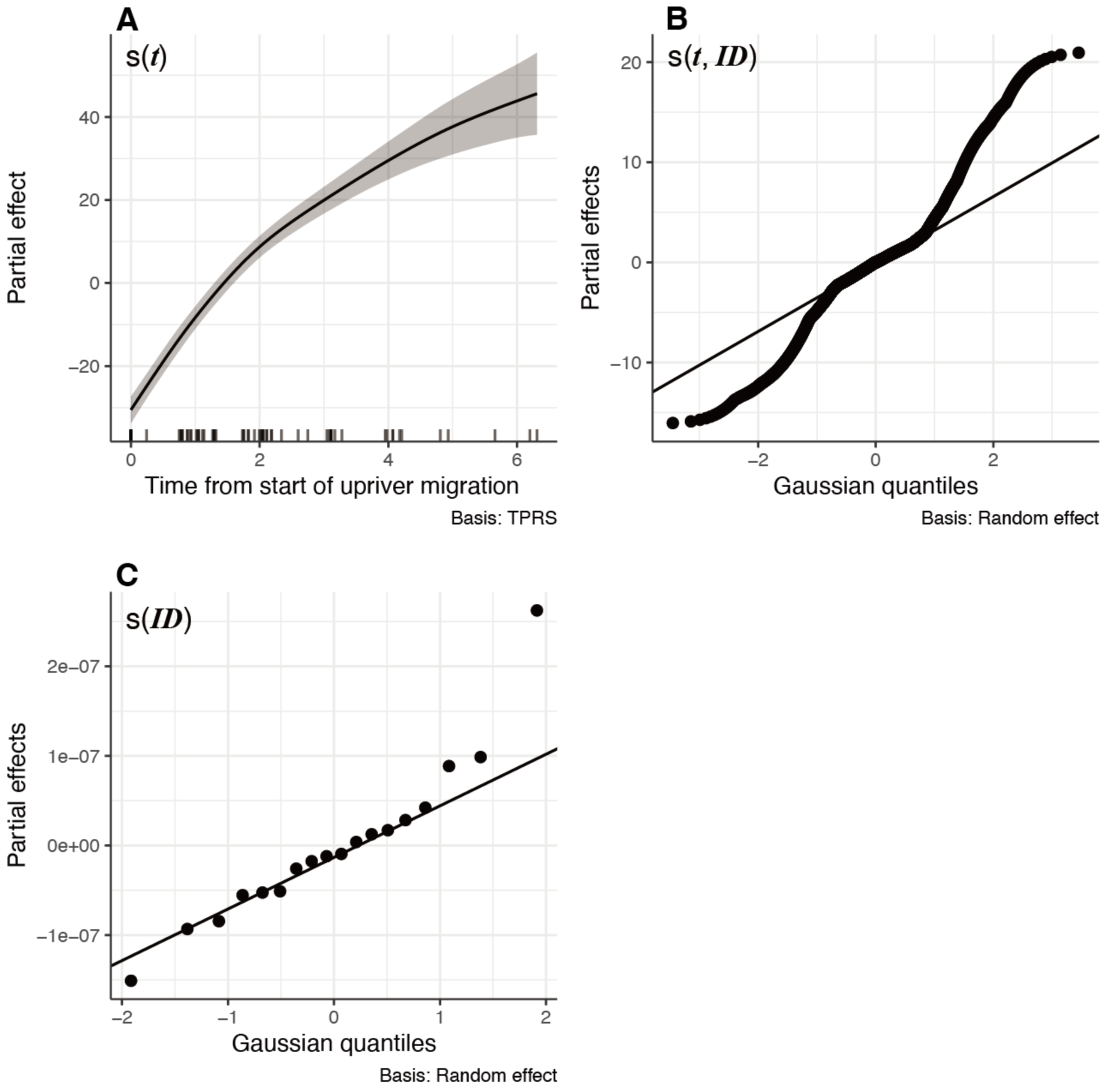


Supplementary Figure 5: Partial effects of smooth terms from the final generalised additive mixed model (GAMM2 in Supplementary Table 5). (A) The estimated smooth effect of time from the start of upriver migration s(*t*) using thin-plate regression splines (TPRS). The shaded area represents the 95% confidence interval. (B) Quantile-quantile plot of the random smooth effect of time by individual s(*t*, *ID*). (C) Quantile-quantile plot of the random effect by individual s(*ID*).

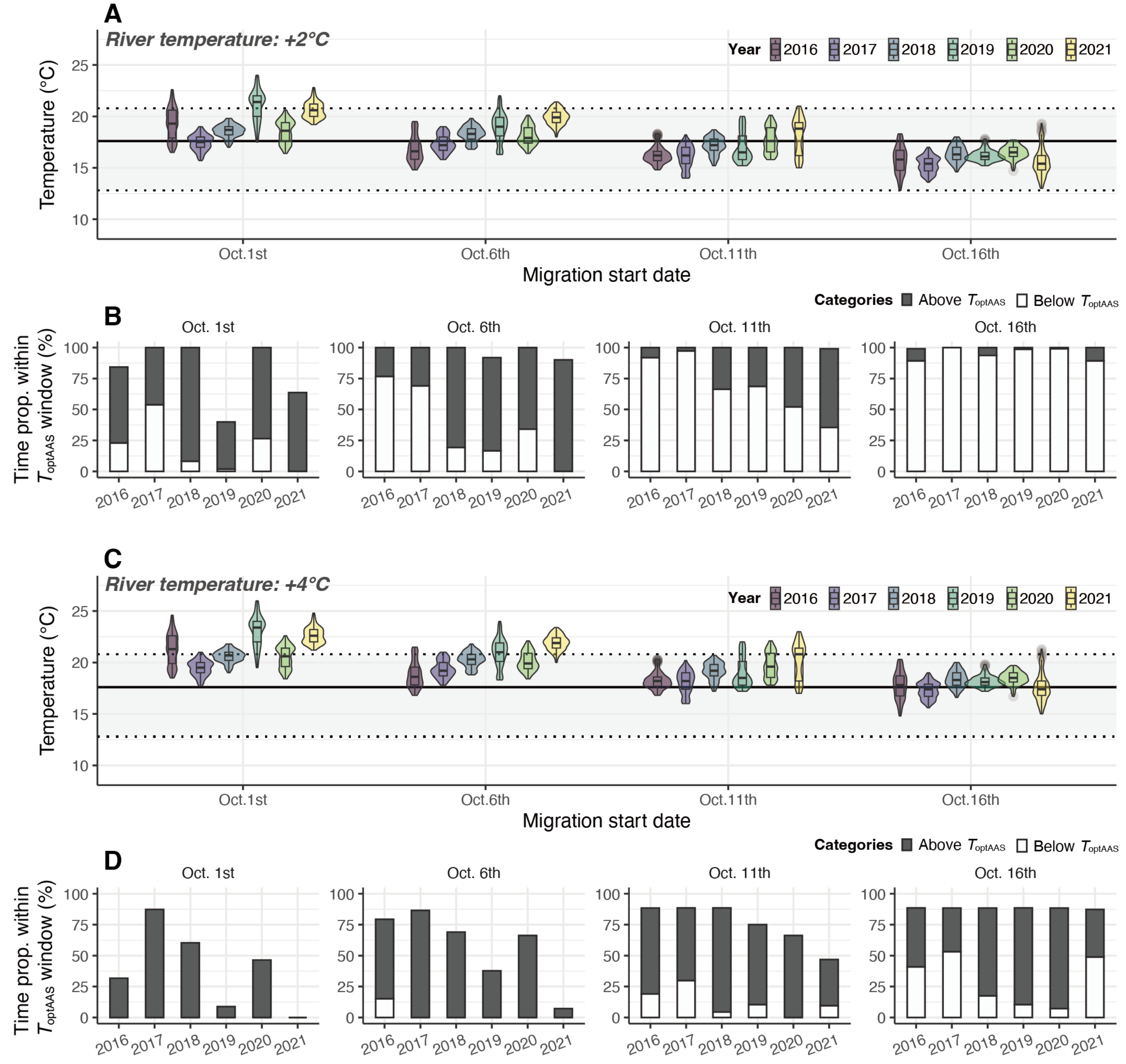


Supplementary figure 6: Estimates of the water temperatures experienced by Kitakami River chum salmon during their upriver migration under elevated temperature conditions and the proportion of time the salmon spent within the optimal temperature window for absolute aerobic scope ($T_{\mathrm{optAAS}}$ window) at that time. Violin and box plots show the estimates of the experienced temperatures from the release point until reaching the spawning site 100 km upstream (a 10-day journey) from each date (1, 6, 11, 16 October) of each year in 2016–2021 under 2°C (A) and 4°C (C) elevated conditions. The horizontal lines in (A) and (C) indicate the optimal temperature for the absolute aerobic scope (black bold line, $T_{\mathrm{optAAS}}$) and the $T_{\mathrm{optAAS}}$ window (dotted lines). The $T_{\mathrm{optAAS}}$ window was defined as the temperature range within 90–100% of the maximum value of AAS. Estimates of the proportion of time spent within the $T_{\mathrm{optAAS}}$ window for each start date of upriver migration under 2°C (B) and 4°C (D) warming conditions. Filled colours denote the proportion of time above $T_{\mathrm{optAAS}}$ (dark colour) and below $T_{\mathrm{optAAS}}$ (light colour).
